# Multiomics analyses revealed the roles of the epithelial–mesenchymal transition and novel early candidate diagnostic biomarkers in neonatal necrotising enterocolitis

**DOI:** 10.1002/ctm2.70793

**Published:** 2026-09-15

**Authors:** Xiao‐Chen Liu, Guo‐Bin Liu, Chen Cheng, Jin Zhu, Fei‐Fan Chen, Ying Ji, Yu‐Ni Zhang, Xiao‐Lin Yan, Qing Wang, Xin‐Yu Li, Qing Ai, Yuan Shi, Yu He

**Affiliations:** ^1^ Department of Neonatology Children's Hospital of Chongqing Medical University Chongqing China; ^2^ National Clinical Research Center for Children and Adolescents’ Health and Diseases Chongqing China; ^3^ Ministry of Education Key Laboratory of Child Development and Disorders Chongqing China; ^4^ Chongqing Municipal Health Commission Key Laboratory of Children's Vital Organ Development and Diseases Chongqing China; ^5^ Department of General and Trauma Surgery Children's Hospital of Chongqing Medical University Chongqing China; ^6^ Department of Pathology, College of Basic Medicine Chongqing Medical University Chongqing China; ^7^ Molecular Medicine Diagnostic and Testing Center Chongqing Medical University Chongqing China; ^8^ Department of Pathology Children's Hospital of Chongqing Medical University Chongqing China; ^9^ Department of Laboratory Children's Hospital of Chongqing Medical University Chongqing China

**Keywords:** biomarkers, dysfunctions, epithelial–mesenchymal transition, neonatal necrotising enterocolitis, proteomics

## Abstract

**Background:**

Necrotizing enterocolitis (NEC) is a severe neonatal gastrointestinal disease with incompletely understood pathogenesis and a lack of reliable early biomarkers. We investigated intestinal molecular alterations associated with NEC and explored candidate diagnostic biomarkers.

**Methods:**

Bulk proteomics of formalin‐fixed paraffin‐embedded intestinal tissues was integrated with spatial proteomics of an index NEC case, single‐cell RNA sequencing, immunohistochemical validation, serum enzyme‐linked immunosorbent assays, and a murine NEC model with pharmacological inhibition of Snail‐mediated epithelial–mesenchymal transition (EMT).

**Results:**

Multiomics analyses indicated layer‐associated epithelial injury, inflammatory activation, and EMT‐like fibroblast/myofibroblast remodelling during NEC. Single‐cell sequencing of six intestinal samples showed reduced epithelial‐cell abundance with increased fibroblast and smooth‐muscle‐cell proportions in NEC. In the murine model, CYD19 treatment was associated with improved intestinal pathology, survival, inflammatory cytokine profiles, and barrier‐related readouts. ANXA2, RPL13, RPL23A, and RPS28 were identified as tissue candidate biomarkers and validated by immunohistochemistry. Serum ANXA2 was higher in NEC than in available non‐NEC controls (AUC, 0.944) and showed exploratory discrimination between medical NEC and highly suspected cow’s‐milk protein allergy; the adjusted AUC was 0.899.

**Conclusions:**

NEC is associated with inflammation‐linked EMT‐like intestinal remodelling and epithelial injury. ANXA2 and selected ribosomal proteins are candidate tissue biomarkers, while serum ANXA2 warrants prospective multicentre validation as a blood‐based diagnostic and risk‐stratification biomarker.

**Key points:**

- NEC was associated with epithelial injury, excessive inflammation, and EMT‐like fibroblast/myofibroblast remodelling, as revealed by integrated bulk proteomics, spatial proteomics, and single‐cell sequencing.
- Pharmacological inhibition of Snail‐mediated EMT in a murine NEC model was associated with improved intestinal injury, inflammatory responses, barrier‐related readouts, and survival.
- ANXA2, RPL13, RPL23A, and RPS28 were identified as candidate tissue biomarkers, while serum ANXA2 showed exploratory potential for NEC diagnosis and risk stratification.

## Graphical abstract

In infants with necrotising enterocolitis (NEC), excessive inflammation and epithelial cell death promote the epithelial–mesenchymal transition (EMT), leading to damage to intestinal barriers and the translocation of bacteria and toxins. In addition, inflamed epithelia release cytokines to strengthen the inflammatory response. Moreover, the EMT promotes the differentiation of myofibroblasts and extracellular matrix deposition, which potentially cause fibrosis after NEC.

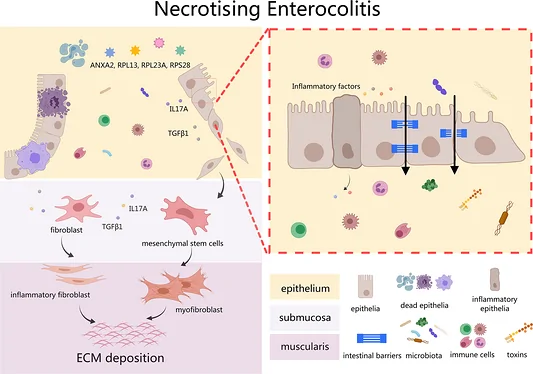

## 1 INTRODUCTION

Necrotising enterocolitis (NEC) is an acute, life‐threatening gastrointestinal disorder in neonates, especially preterm infants, with an insidious onset and atypical early symptoms. Despite medical advances in neonatology, the incidence of NEC has been increasing among very‐low‐birth‐weight and extremely low‐birth‐weight neonates. Studies have shown a high incidence of NEC (up to 28%) among neonates born at 23 weeks of gestation.^1^, ^2^, ^3^, ^4^ The mortality rate can reach 24.1%^5^ ^–^ ^7^ and is even higher in infants requiring surgery.^8^ Survivors typically suffer long‐lasting consequences, including intestinal stenosis, short bowel syndrome after surgery and neurological sequelae caused by exaggerated immune responses, with a significant reduction in quality of life.^9^, ^10^, ^11^ However, the aetiology and pathogenesis of NEC remain unclear at present, and the diagnosis depends mainly on the clinical presentation and imaging features, which frequently result in a delayed diagnosis and poor outcomes. Treatments, including fasting, gastrointestinal decompression or empirical antibiotic administration, are non‐specific and non‐targeted interventions.^12^, ^13^ Therefore, an investigation of the aetiology and pathogenesis of NEC is necessary for early diagnosis, the development of specific treatments and potential improvement of outcomes in infants with NEC.

In recent years, many studies have focused on exploring the pathogenesis and biomarkers of NEC. The main goal of these studies was to investigate clinical body fluids and faecal samples that are released into the intestinal lumen or peripheral circulation after intestinal injury, for which little direct evidence is available and changes are frequently detected in individuals with other infectious diseases or intestinal injury disorders.^14^ Studies with animal intestines or a small number of intestinal samples from infants with NEC, which have low specificity and credibility, have shown that the majority of associated phenomena may not accurately and directly reflect the pathological and physiological processes and progression of NEC.^15^, ^16^, ^17^ Currently, with advances in protein extraction, proteins can be extracted from archived formalin‐fixed paraffin‐embedded (FFPE) samples and analysed.^18^, ^19^ An emerging form of proteomics, spatial proteomics, can not only provide details of protein expression but also retain spatial information in tissues, enabling researchers to explain pathological progression in samples with both normal and lesional areas^20^, ^21^; this method was recognised as a method of the year in 2024 by *Nature Methods*.^22^ Therefore, we aimed to characterise alterations in protein expression and function and to identify potential early diagnostic biomarkers by performing bulk and spatial proteomic analyses from FFPE intestinal tissues samples from infants who underwent NEC surgeries and single‐cell sequencing. Immunohistochemical (IHC) staining and enzyme‐linked immunosorbent assays (ELISAs) were used to validate the efficacy of the early diagnostic biomarkers. Our aim was to provide novel signatures for the early diagnosis and targeted treatment of NEC.

## 2 SUBJECTS AND METHODS

### 2.1 Patients

Infants from the Neonatal Diagnosis and Treatment Center of Children's Hospital of Chongqing Medical University were enrolled in this study. The cohort for proteomic and single‐cell sequencing analyses included infants who were admitted between July 2021 and June 2023; samples from this cohort were also used for the internal validation IHC cohort. The infants used for external validation IHC staining cohort were enrolled between July 2023 and June 2025. Infants enrolled in the ELISA cohort for the validation of serum biomarkers were admitted between July 2024 and June 2025. This study was approved by the Ethics Committee of the Children's Hospital of Chongqing Medical University (No. 2023‐540, 2024‐395) and was prospectively registered in the China Clinical Trial Center (Pid: 283287). Infants in proteomic and IHC staining cohort were enrolled retrospectively and infants in single‐cell sequencing analyses and ELISA cohort were enrolled prospectively with parental consent obtained.

### 2.2 Sample size estimation

The sample sizes of the external IHC cohort and ELISA cohort were determined with G*Power 3.1 software,^23^ and the parameter settings were as follows: two‐tailed test, *α* = .05; Cohen's effect size, *d* = 1.0 (based on the difference in biomarker expression in the proteomics cohort); and power, (1 − *β*) = .8.^24^ The calculations revealed that at least 17 infants were needed per group in the external IHC cohort, and at least 13 infants in the C group, 25 infants in the N group (including 18 infants in M‐NEC group), and 18 infants in the HS‐CMPA group were needed in the ELISA cohort. Therefore, we ultimately included 40 infants in the external IHC cohort (20 per group) and 133 (42 in the C group, 65 in the N group and 26 in the HS‐CMPA group) in the ELISA cohort.

### 2.3 Inclusion criteria

In the proteomic, single‐cell sequencing and IHC cohorts, neonates who were diagnosed with NEC according to the modified Bell staging criteria^25^, ^26^ were included in the N group. In the proteomics cohort and single‐cell sequencing, neonates diagnosed with omphalocele^27^ were included in the control group (C group), neonates diagnosed with intestinal atresia^28^ were included in the C group of the IHC staining cohort.

Infants with bloody stools were included in the ELISA cohort, and serum samples for routine peripheral blood testing within 12 h after enrolment were collected. Neonates who were ultimately diagnosed with NEC were included in the N group, and those whose imaging or clinical manifestations deteriorated after medical treatment and who underwent surgical treatment after an evaluation by two senior surgeons were included in the surgical‐NEC group (S‐NEC). Neonates who recovered after medical treatment were included in the medical‐NEC group (M‐NEC). Neonates who were highly suspected to have cow's‐milk protein allergy (CMPA) with an improvement in the clinical symptoms after receiving a diagnostic elimination diet^29^ were included in the HS‐CMPA group. To address the difficulties associated with obtaining samples from completely healthy infants, infants hospitalised for hyperbilirubinemia were included, and those without any digestive tract symptoms, signs of infection, organ dysfunction or worsening conditions during hospitalisation were included as the available non‐NEC clinical control in the C group, and the corrected gestational age of the enrolled infants was matched with that in the N group. Residual serum samples from routine peripheral blood testing within 48 h before discharge were collected.

### 2.4 Exclusion criteria

The exclusion criteria were as follows: (1) infants diagnosed with other congenital gastrointestinal malformations, such as megacolon; (2) infants with genetic metabolic disorders; (3) infants whose remaining blood samples were insufficient for testing in the ELISA cohort; and (4) infants whose parents did not consent to inclusion.

### 2.5 Data collection

Demographic data such as sex, gestational age, birth weight and perinatal high‐risk factors, including gestational diabetes, gestational hypertension and chorioamnionitis, were collected. Disease outcomes such as mortality, hospital stay and complications, including intestinal stenosis, septic shock and malnutrition, were also recorded. Septic shock was defined as sepsis with tissue hypoperfusion and cardiovascular dysfunction.^30^ Malnutrition was evaluated at 1 year of age and defined as a weight below the average of infants at the same age and sex by more than two standard deviations.^31^

### 2.6 Animals

The animal experiments were approved by the Animal Ethics Committee of Children's Hospital of Chongqing Medical University (No. CHCMU‐IACUC20240412010). Specific pathogen‐free C57BL/6 mice were obtained from Chongqing Medical University and housed at 20–25°C on a 12:12‐h light–dark cycle with unrestricted food and water. On the seventh day after birth, newborn mice were used for NEC induction as previously described.^32^ Seven‐day‐old mice were hand‐fed high‐sodium milk (2 g of Esbilac Puppy and 3.3 g of Similac Advance Replacer in 10 mL of drinking water) every 4 h for 3 days. They were subjected to asphyxia (100% nitrogen for 90 s) followed by cold stress at 4°C for 10 min three times a day for 3 days. The control mice were housed with their mothers and received no intervention. Lipopolysaccharide (LPS) was given once a day (5 mg/kg; L2630, Sigma) in NEC + LPS and NEC + LPS + CYD19 group. The mice were intraperitoneally injected with the Snail inhibitor CYD19 (15 mg/kg; HY‐144315, MedChemExpress) every day during NEC modelling in NEC + CYD19 and NEC + LPS + CYD19 group. Before sacrifice, the mice were allowed to relax for 12 h after feeding, and the intestines were harvested and stored at −80°C for subsequent experiments.

## 3 METHODS

### 3.1 Sample preparation and selection of the target area for the proteomic analyses

For the bulk proteomics,^33^ five sections (4 µm thick and 10 mm^2^ in area containing more than 80% of the total area of the target tissue types) from FFPE blocks for each infant were deparaffinised with xylene, washed within an ethanol gradient and scraped into tubes. For the spatial proteomics, the sections were accurately evaluated through haematoxylin and eosin (HE) staining to distinguish the inflamed areas from the relatively normal areas. Expansion microscopy was used to expand and stain the sections.^34^ Following denaturation in expansion and deformation buffers, the sections were immersed in 50% and 100% methanol solutions. Afterwards, it was stained with Coomassie Brilliant Blue and immersed in balance solution. A laser capture microdissection (LCM) system was used to selectively cut and eject energy on the designated area, and the ejected tissue was collected in specially designed collection tubes.^35^

### 3.2 Protein extraction from FFPE sections and trypsin digestion

The samples were lysed, denatured and enzymatically digested using the FFomic process.^36^ For the spatial proteomics, the samples were dehydrated with acetonitrile, and the gel blocks were rehydrated with 50 mM ammonium bicarbonate. Samples for bulk and spatial proteomics were all lysed in Tris(2‐carboxyethyl) phosphine hydrochloride buffer supplemented with protease and phosphatase inhibitors at 99°C for 30 min. After cooling, trypsin was introduced, and the samples were digested for 18 h at 37°C. Afterwards, 10% formic acid was added and the samples were vortexed and centrifuged at high speed. Extraction buffer with  .1% formic acid in 50% acetonitrile was added to extract the supernatant, and the supernatant was subsequently transferred to a new tube for drying using a SpeedVac. The digested amplicons were quantified to determine the peptide concentration, and the peptides were loaded onto the instrument with the same peptide mass.

### 3.3 Label‐free‐based liquid chromatography–mass spectrometry/mass spectrometry (LC–MS/MS) analysis for bulk proteomics

Peptides were analysed with an OE480 Hybrid Quadrupole‐Orbitrap mass spectrometer (Thermo Fisher Scientific) connected to a high‐performance liquid chromatography system (EASY nLC 1200, Thermo Fisher Scientific). Mobile phase A was a  .1% formic acid solution, and mobile phase B was a mixture of  .1% formic acid and 80% acetonitrile. First, the chromatographic column was equilibrated with 100% mobile phase A, and then the enzymatically digested peptides from the samples were automatically delivered to the loading column and further separated through the analytical column at a flow rate of 600 nL/min before being injected into the mass spectrometer. The mass spectrometer was used in positive ion mode with a scan range of 300–1400 *m*/*z*, a primary spectrum resolution of 120 000 at 200 *m*/*z*, an automatic gain control target of 3e6, a maximum injection time of 80 ms and a dynamic exclusion time of 40.0 s. The mass‐to‐charge ratios of the peptides and peptide fragments were collected with a full scan time of 1.5 s of the fragment spectra and a normalised collision energy of 30%, an isolation window at 1.6 *m*/*z* and a secondary mass spectrum resolution of 7500 at 200 *m*/*z* with high‐energy collision dissociation.

### 3.4 Data‐independent acquisition–mass spectrometry (DIA–MS) analysis for spatial proteomics

The samples were separated using a method similar to that mentioned above. After chromatographic separation, the peptide samples were subsequently introduced into a Q Exactive HF‐X mass spectrometer (Thermo Fisher Scientific) in positive ion mode. The mass range was 300–1400 *m*/*z*, with a primary spectrum resolution of 60 000 at 200 *m*/*z*, an automatic gain control target of 3e6 and a maximum injection time of 20–30 ms. The mass–charge ratios of the peptides and peptide fragments were collected with 30 DIA scans per scan, with a collision energy of 27%, a varied isolation window and a secondary mass spectrum resolution of 7500–15 000 at 200 *m*/*z* with high‐energy collision dissociation.

### 3.5 Quantification of proteins

The iProteome platform was used for protein quantification. Briefly, based on the peptide identification information, the extracted‐ion chromatogram (XIC) was extracted by searching against the MS1, and the abundance was estimated by calculating the area under the extracted XIC curve. Protein abundance was calculated with the non‐redundant peptide list to parsimony principle, with a traditional label‐free, intensity‐based absolute quantification algorithm. Matching between runs was enabled to transfer the identification based on accurate mass and retention time after retention time alignment. A dynamic regression model was established, and based on the correlation coefficient *R* ^2^, linear or quadratic regression functions were selected to estimate the retention times of the corresponding hidden peptides and validate the presence of XIC. The normalised abundance of a specific protein was subsequently calculated by defining it as a fraction of the total protein.^37^, ^38^, ^39^

### 3.6 Single‐cell sequencing

Fresh intestinal samples were washed with cell culture medium, incubated with 1 µg/mL DNase and 100 µg/mL collagenase A in the same medium overnight and dissociated with a gentleMACS Octo Dissociator with heating (Miltenyi Biotec, 130‐134‐029). The tissues were then filtered through a 70‐µm nylon mesh cell strainer to prepare a single‐cell suspension. Live cells were enriched with a dead‐cell removal kit and a MACS cell separation system (Miltenyi Biotec, 130‐090‐101) and counted. The library was prepared with the Chromium Next GEM Single‐Cell 5′ kit (10× Genomics, PN‐1000020) and sequenced on the Illumina NovaSeq 6000 platform (Illumina). The raw sequencing reads were aligned to the GRCh38 reference genome with CellRanger.

### 3.7 Histological scoring, immunohistochemical staining and immunofluorescence (IF) staining

Murine terminal ileal tissues were fixed overnight with 4% paraformaldehyde, embedded in paraffin, cut into 4 µm sections and stained with HE. After scanning, three fields from each sample were randomly selected and scored in a double‐blind manner as proposed by Dvorak et al.^40^: 0, no injury; 1, mild separation of the lamina propria and/or submucosa; 2, moderate separation of the lamina propria and/or submucosa; 3, severe detachment of the lamina propria and/or submucosa or villi; and 4, villus detachment and necrosis.

The FFPE sections were deparaffinised with xylene and then hydrated in an ethanol gradient. After hydration, antigen retrieval was performed by heating the sections in a citrate solution or triethylenediamine‐ethylenediaminetetraacetic acid solution in a microwave. Endogenous peroxidase blocker (Beyotime, P0100A) and  .3% BSA (Solarbio, A8020) were subsequently used for quenching and blocking.

For IHC staining, the sections were incubated overnight at 4°C with the following primary antibodies: anti‐Snail (1:800; GB11260‐100, Servicebio), anti‐ANXA2 (1:2000; Ab41803, Abcam), anti‐RPS28 (1:100; 14796‐1‐AP, Proteintech), anti‐RPL13 (1:50; 11271‐1‐AP, Proteintech) and anti‐RPL23A (1:100; A4086, ABclonal). Afterwards, the sections were incubated with a goat anti‐rabbit secondary antibody (G1312, Servicebio) for 30 min at 25°C. After diaminobiphenylamine staining, the sections were counterstained, mounted and examined under an optical microscope with three random fields of view selected per sample at 40× magnification to score the IHC staining. The positive area and staining intensity were weighted for H‐scores by at least two individuals who were blinded to the groups, including a professional pathologist, and the average H‐scores of the three views were recorded for each sample.

For IF staining, the sections were incubated with anti‐E‐cadherin (1:1000; AFRP0022, Aifang) and anti‐Vimentin (1:1000; AFRP0062, Aifang) antibodies overnight and then incubated with an HRP‐conjugated goat anti‐rabbit secondary antibody for 30 min at 25°C, tyramide signal amplification fluorescent dyes (AFIHC024, Aifang) for 10 min and DAPI for 10 min. Images were captured with a fluorescence scanner (KF‐FL‐020, KFBIO), and three random fields of each sample were selected for scoring.

### 3.8 Quantitative real‐time PCR (RT‐PCR)

Total RNA was extracted from mouse intestines and purified with an RNAiso kit (Accurate Biotechnology, AG21023), and the OD260/OD280 ratios were determined to be between 1.8 and 2.2. Evo M‐MLV RT Premix for qPCR (Accurate Biotechnology, AG11734) was used for reverse transcription, and a SYBR Green Premix Pro Taq HS qPCR Kit (AG11733, Accurate Biotechnology) was used for amplification. SYBR green‐based RT‐qPCR was performed on a Bio‐Rad CFX96 system, gene expression was analysed with normalisation to β‐actin expression, and relative mRNA expression levels were quantified with the 2^(−ΔΔCt)^ method. The sequencing information is provided in Table S1.

### 3.9 Enzyme‐linked immunosorbent assay

Intestinal tissues homogenised in PBS and blood samples were centrifuged at 3000 rpm for 10 min, and the supernatant was stored at −80°C. In accordance with the instructions of the kits for Il‐6 (CME0006, 4A BIOTECH), Il‐10 (CME0016, 4A BIOTECH), Tnf‐α (CME0004, 4A BIOTECH), ANXA2 (JL52475, Jonlnbio), CALP (JL19401, Jonlnbio), FABP2 (JL44346, Jonlnbio), IL‐6 (JL14113, Jonlnbio) and IL‐8 (JL19291, Jonlnbio), the samples were sequentially incubated with a biotinylated antibody and horseradish peroxidase–enzyme conjugates. Tetramethylbenzidine substrate and stop solution were added before the absorbance was measured at a wavelength of 450 nm.

### 3.10 Data analysis

The results of the bioinformatic and figure drawings for the proteomics and single‐cell sequencing were performed with R (version 4.3.1, New Zealand) and Python (version 3.9.7). The proteins were subjected to BLAST searches against the online Kyoto Encyclopedia of Genes and Genomes (KEGG) database (https://www.kegg.jp/).^41^, ^42^ Gene set enrichment analysis (GSEA) was performed with gseapy (Python) after enriched gene sets were obtained from the Molecular Signatures Database (MSigDB; https://www.gsea‐msigdb.org/gsea/msigdb).^43^ The single‐cell and unique molecular identifier count matrices were imported into R for further analysis with the Seurat package (version 5.1.0).^44^ Cells were filtered with gene counts between 200 and 4000, as well as genes that appeared in more than three cells with fewer than 20% of genes from the mitochondrial genome. Doublet cells were removed with DoubletFinder, and gene expression was determined with the LogNormalise function in Seurat.^45^ Principal component analysis (PCA) was performed (npcs = 20), followed by uniform manifold approximation and projection (UMAP) analysis. Neighbours were identified with PCA components, and clusters were defined with a resolution of  .1. Cell types were annotated based on the marker gene expression. GSEA was performed with the Hallmark function in the R package clusterProfiler (version 4.12.6).^46^ We used the R package Monocle3 to align cells with the developmental trajectory.^47^, ^48^ Clinical data from the enrolled infants were analysed with SPSS statistical software (version 24). IHC staining scores and biomarker concentrations were analysed with ImageJ (version 1.54) and GraphPad Prism (version 9.0). Normally distributed measurement data are presented as the means ± S.D.s. and were analysed using Student's *t*‐test. Non‐normally distributed measurement data are presented as medians (interquartile ranges [IQRs]) and were analysed using the Wilcoxon rank‐sum test. Count data were analysed using Fisher's exact test. Comparisons of pathways, cells or proteins between different groups were analysed using Student's *t*‐test. The correlations between protein expression and clinical features were analysed by calculating Pearson's correlation coefficients. A significance level of *p* < .05 was considered to indicate statistical significance. The graphical abstract and flowcharts were created with MedPeer (medpeer.cn).

## 4 RESULTS

**FIGURE 1:**
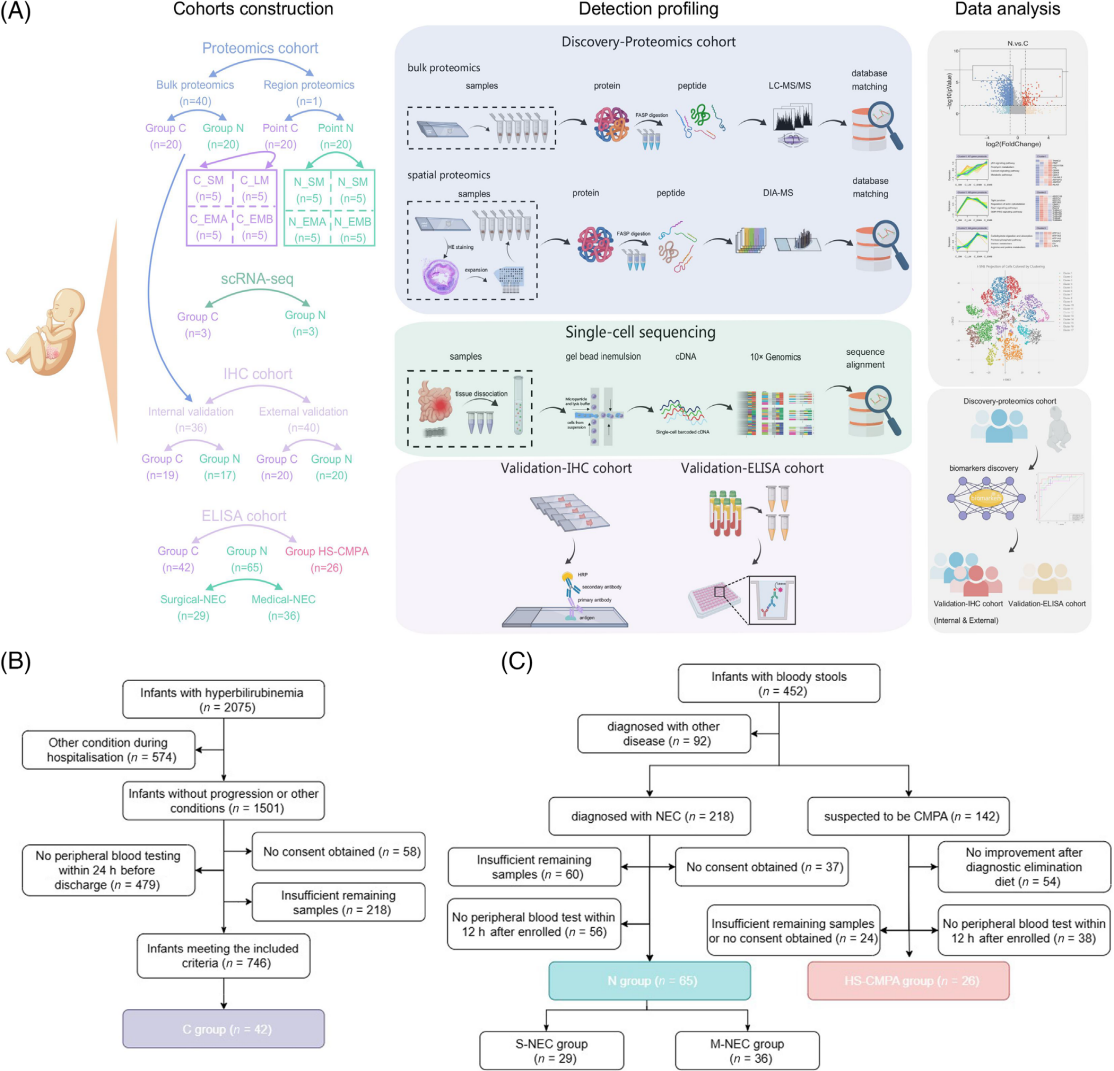
The experimental design involved the collection of samples. (A) Cohorts construction and data acquisition and processing of the study. (B) Flowchart of the enrolment of infants in C group of enzyme‐linked immunosorbent assay (ELISA) cohort. (C) Flowchart of the enrolment of infants in N and HS‐CMPA groups of ELISA cohort.

A total of 213 infants were included in this study. Among these patients, 40 were included in the proteomics cohort to characterise NEC, with one randomly chosen for the spatial proteomics to clarify the pathological process and six undergoing single‐cell sequencing, 40 were included in the IHC staining cohort for external validation, and 133 were included in the ELISA cohort. Samples from 36 of the 40 infants subjected to bulk proteomics were included in the IHC staining cohort for internal validation, and four were excluded because of the unavailability of FFPE samples (Figures 1A and S1). In the ELISA cohort, 2075 infants were included in the C cohort, and 452 were included in the bloody stool cohort; after the exclusion criteria and matching between groups were applied, 42 infants were ultimately included in the C group, 26 were included in the HS‐CMPA group and 65 were included in the N group, including 29 surgically treated and 36 medically treated (Figure 1B,C). In the bulk proteomics and IHC cohorts, the neonates with NEC had younger gestational ages and lower birth weights and Apgar scores (*p *< .05) and in the ELISA cohort, basic demographic differences between C and N group were not significant (*p *> .05), except for longer hospital stays (*p *< .05) and the gestational age of the infants in HS‐CMPA group was younger, the birth weight was lower and the hospital stay was shorter than infants in the M‐NEC group (*p *< .05), suggesting more severe disease conditions in the NEC groups (Table S2).

### 4.1 The hierarchical structure and functional transitions in the epithelium and muscularis layer became disordered during NEC progression

**FIGURE 2:**
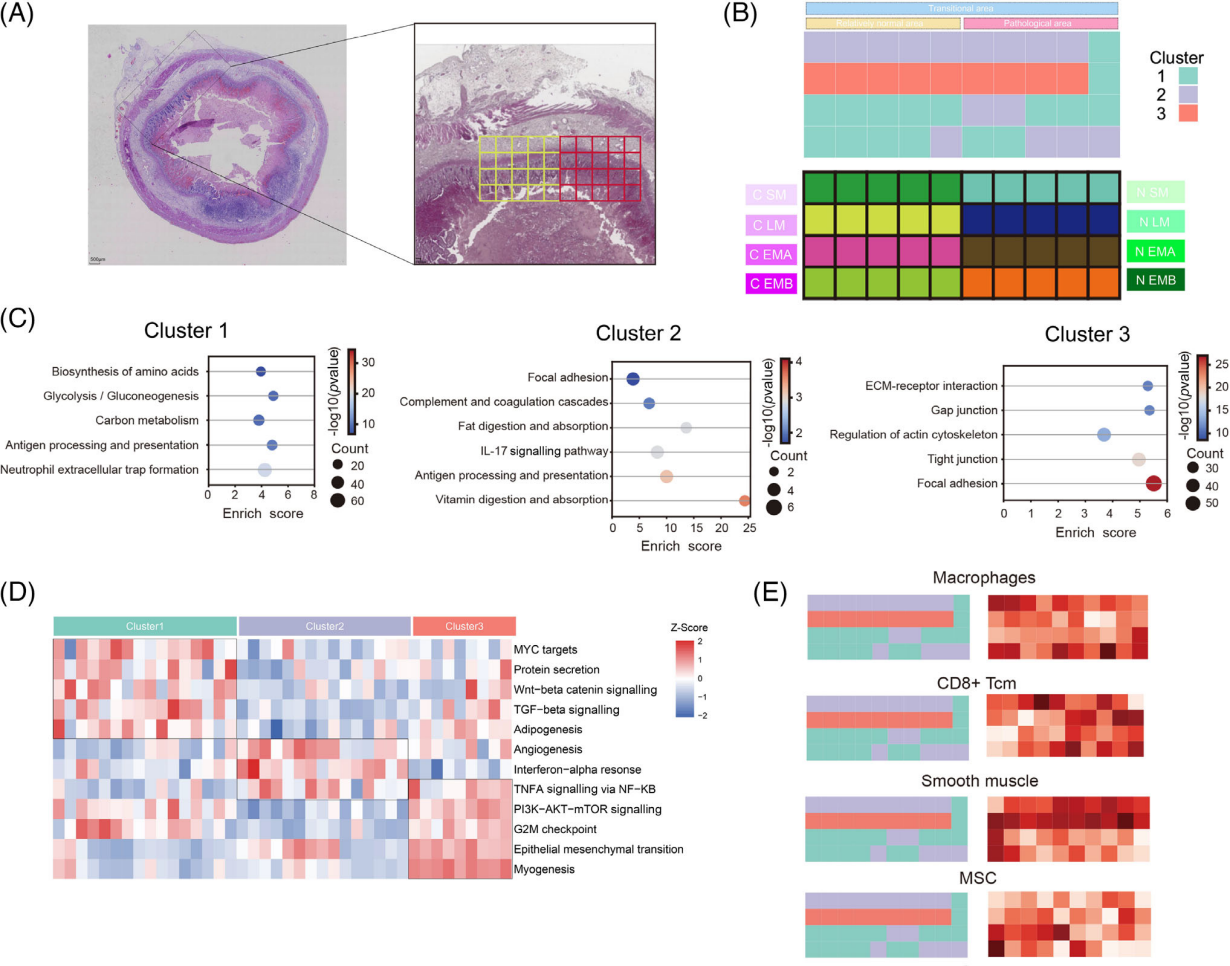
Characteristics of clusters resulting from the spatial proteomics and dimensionality reduction. (A) Regions selected for protein extraction and identification by haematoxylin and eosin (HE) staining. (B) Projection of clusters onto transitional and necrotic regions and grouping within transitional regions. (C) Kyoto Encyclopedia of Genes and Genomes (KEGG) enrichment analysis of proteins identified within each cluster. (D) Gene set variation analysis (GSVA) of pathway activity within each cluster. (E) Mapping of predominant cell types within each cluster using XCell scores.

In accordance with previous studies,^49^, ^50^, ^51^ a spatial proteomic analysis of the transitional regions, including relatively normal and pathological regions from different areas of the FFPE samples from an infant with NEC, was performed to ensure the same microenvironment and genetic background and to elucidate the progression of pathogenesis and spatial‐specific molecular alterations associated with NEC, with a focus on the protein expression and functional characteristics of different layers of the intestinal wall. After a microscopic expansion of selected areas, 40 areas from the epithelial, submucosal and muscularis layers were chosen separately for protein extraction and identification (Figure 2A) and then spatially projected (Figure 2B). PCA indicated the stabilisation of the three components (Figure S2A). A graph‐based clustering algorithm was subsequently used to reduce the dimensionality of the clustering, resulting in three relatively independent clusters, which were spatially projected (Figures S2B and 2A).

KEGG enrichment analysis and gene set variation analysis (GSVA) were conducted to characterise the three clusters. Cluster 1, which was primarily located in the epithelial area, was associated with metabolic pathways and inflammation‐related processes (Figure 2C). GSVA revealed links to protein secretion, adipogenesis and cell proliferation (MYC targets; Figure 2D). Cluster 2, which was located predominantly in the muscularis layer, was linked to focal adhesion, fat digestion and absorption, and inflammatory pathways such as IL‐17 signalling (Figure 2C). GSVA highlighted angiogenesis and the interferon‐alpha response (Figure 2D). Cluster 3, which was located in the submucosa, was involved in barrier functions such as gap and tight junctions, cell migration via focal adhesion and extracellular matrix (ECM)–receptor interactions, and actin cytoskeleton regulation (Figure 2C). GSVA revealed key functions related to the epithelial–mesenchymal transition (EMT), myogenesis and pathways regulating inflammation (Figure 2D). Based on the protein expression in the transitional area, we classified cluster 1 as epithelium‐like, cluster 2 as muscularis‐like and cluster 3 as submucosa‐like (Figure S2C–E). However, in the necrotic area, epithelium‐like functions were observed in the submucosa and muscularis layer, whereas muscularis‐like functions appeared in the epithelium, suggesting potential transition disorders between the epithelium and muscularis layer.

To investigate this transition, we examined proteins involved in key pathways and cell types across the three clusters. Cluster 2, which was associated with muscularis‐like functions, was prominently observed in the epithelium of the necrotic area. This cluster was linked to inflammatory responses such as IL‐17 signalling, suggesting increased inflammation in the necrotic area of the epithelium. The XCell (https://xcell.ucsf.edu)^52^ analysis revealed increased numbers of inflammatory immune cells, such as macrophages and central memory CD8^+^ T cells, in the necrotic epithelium. In the adjacent cluster 3, which had a submucosa‐like function, proteins involving in the EMT pathway were highly expressed (Figures 2D,E and S2F), suggesting that the activation of EMT pathway in NEC intestinal tissue might be correlated with excessive inflammatory pathways. The coactivation of inflammatory responses and the EMT may promote the functional disorders of intestinal wall tissue. Notably, cluster 1, with epithelium‐like functions, appeared in the submucosa and muscularis layer (Figure 2B). Neutrophil extracellular trap formation indicated an inflammatory response in the submucosa and muscularis layer, alongside the initial EMT regulators Wnt‐beta catenin and TGF‐beta signalling pathways (Figures 2D and S2F). Cluster 1 had a decreased number of MSCs, and cluster 2 had increased numbers of smooth muscle cells (SMCs) upon projection (Figure 2E). These findings suggest that the activated EMT and its subsequent effects such as a loss of connection of epithelial cells may contribute to transition disorders by linking excessive inflammation, barrier repair imbalance and intestinal dysfunction in NEC progression.

### 4.2 Spatial proteomics of an index NEC case suggested layer‐associated epithelial injury and EMT‐like mesenchymal remodelling

We investigated the roles of transition disorders and their effects on NEC progression by analysing the molecular characteristics of necrotic and transitional tissues. The samples were categorised into groups N and C based on the severity of tissue necrosis and further classified into four subgroups: muscularis (SM), submucosa (LM), proximal epithelial (EMA) and distal epithelial (EMB; Figure 2B).

**FIGURE 3:**
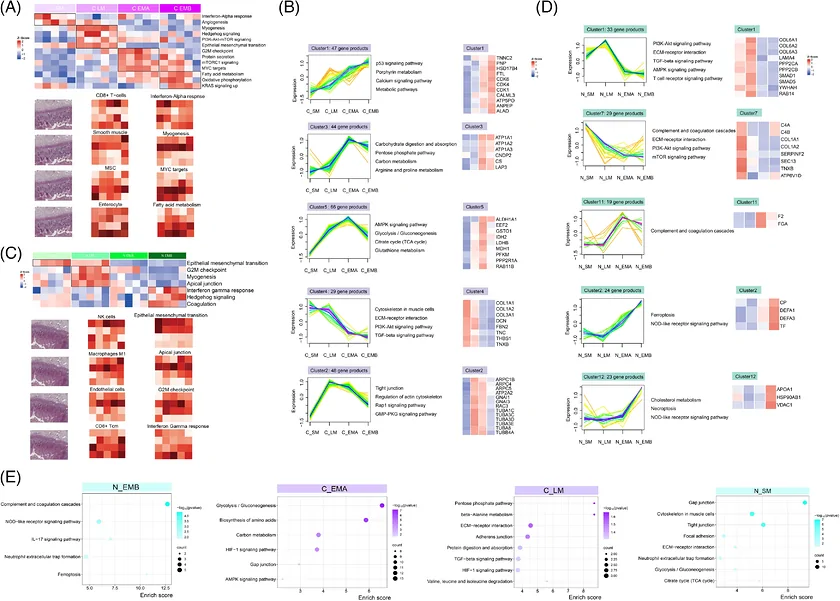
Alterations across distinct tissue hierarchies in the two groups and analysis of differences in the results of the spatial proteomic analysis between the two groups. (A) Gene set variation analysis (GSVA) of pathways identified in each subgroup categorised by tissue type in the C group and spatial mapping of cell types and pathways within each subgroup in the C group. (B) Variations in key pathways and proteins across tissue hierarchies in the C group. (C) GSVA of pathways in each subgroup classified by tissue type in the N group and spatial representation of cell types and pathways within each subgroup in the N group. (D) Alterations in the principal pathways and proteins across tissue hierarchies in the N group. (E) Kyoto Encyclopedia of Genes and Genomes (KEGG) analysis of upregulated pathways in the different areas of tissues from the N group.

We first analysed the four subgroups within the C and N groups separately. Each subgroup of the C group exhibited distinct functional characteristics. The EMB subgroup was primarily associated with enterocytes and involved in energy metabolism. The EMA subgroup was characterised by MSCs and linked to cell proliferation and metabolic regulation. The LM subgroup was associated with SMCs and involved in muscle proliferation with the EMT also playing a significant role. The SM subgroup was characterised by the presence of CD8^+^ T cells and immune responses (Figure 3A). Proteins that exhibited similar changes were subsequently grouped into 12 clusters to delineate functional changes across tissue areas in normal tissue (Figures S3A and 3B). However, in the N group, the EMA and EMB groups lost their epithelial cell characteristics. The EMA group primarily exhibited endothelial features, possibly indicating mesenchymal transformation, whereas the EMB group showed increased interferon gamma and inflammatory responses, with central memory CD8^+^ T cells becoming predominant. The LM group displayed increased muscle formation and barrier function as well as macrophage M1 infiltration. In contrast, the SM group was associated with the EMT and an abundance of NK cells (Figure 3C). Proteins were categorised into 12 clusters (Figure S3B), with the EMA and EMB groups diverging functionally from the C group, shifting from metabolism to inflammatory responses and cell death pathways, including complement and coagulation cascades, ferroptosis, necroptosis and nucleotide‐binding oligomerization domain (NOD)‐like receptor signalling (Figure 3D). In clusters 1 and 7, the LM and SM groups maintained ECM–receptor interactions and related signalling pathways, whereas inflammatory responses and T‐cell receptor signalling, emerged in the LM and SM groups of the N group. These findings suggest that NEC onset is initiated by epithelial cell inflammation and death, which might be associated with the EMT and increased ECM–receptor interactions in the muscularis layer.

To elucidate the roles of inflammation‐associated EMT in NEC, we compared protein expression with each subgroup of groups C and N using spatial proteomics. In the epithelium, decreased metabolism and increased inflammatory responses were observed. Specifically, in the distal epithelium, the activity of the IL‐17 signalling pathway which is an important trigger of the EMT was upregulated in the N group. Conversely, in the proximal epithelium, pathways related to energy metabolism and metabolic regulation pathways were downregulated in the N group, indicating the loss of epithelial cells. In the submucosa, the IL‐17 signalling pathway was also upregulated. Conversely, pathways involved in cell migration, polarity regulation and matrix formation, including the core regulatory pathway of the EMT, TGF‐beta signalling were downregulated, indicating a repair disorder. This imbalance of increased inflammation and impaired repair may contribute to the progression of necrosis. In the muscularis layer, a marked increases in cell migration, matrix formation, barrier function and muscle generation were observed (Figures 3E and S3C). Overall, our findings reveal not only the transition from the epithelium but also an imbalance between inflammation and repair weakening by EMT correlated with excessive inflammation. This imbalance progresses to the muscularis layer and might contribute to the deterioration of NEC.

### 4.3 The bulk proteomic analysis revealed the potential effect of the inflammation‐correlated EMT on infants with NEC

**FIGURE 4:**
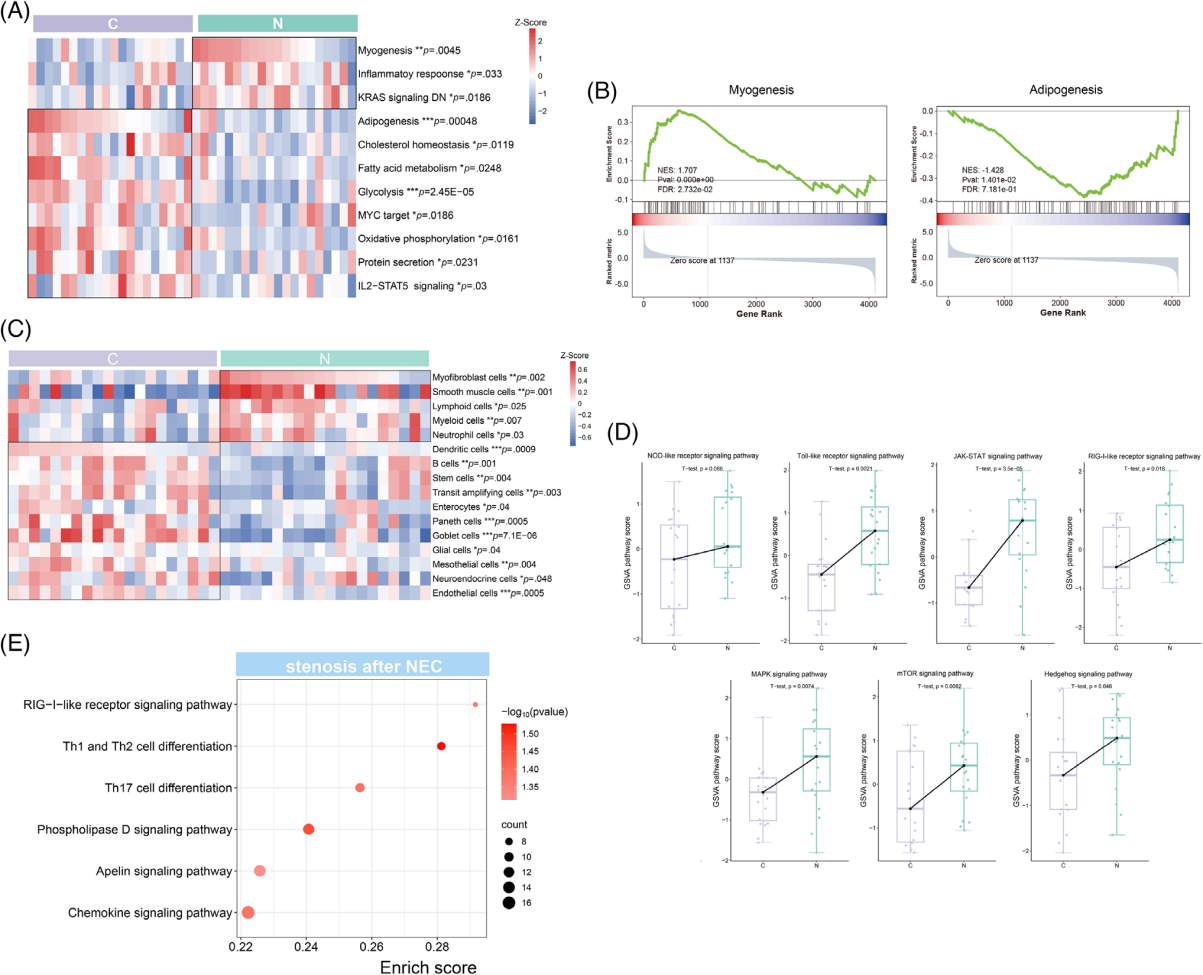
Results of the bulk proteomic analysis and relationships between protein levels and clinical indicators. (A) Gene set variation analysis (GSVA) of pathway activity based on samples from the C and N groups. (B) Gene set enrichment analysis (GSEA) of pathways enriched with differentially expressed proteins between the C and N groups. (C) Heatmap illustrating predominant cell types determined based on the XCell scores. (D) Kyoto Encyclopedia of Genes and Genomes (KEGG) enrichment analysis of inflammation‐related signalling pathways between the two groups. (E) KEGG enrichment analysis of upregulated proteins in individuals with stenosis after necrotising enterocolitis (NEC).

Given the potential role of the inflammation‐associated EMT in NEC pathogenesis, we wanted to explore its effect and relationship with the typical phenotype and prognosis of patients with NEC. Therefore, a bulk proteomic analysis based on NEC and normal intestinal FFPE samples was performed. Pathways were explored with GSVA and GSEA, and the results revealed the upregulation of myogenesis and the inflammatory response and downregulation of metabolism in the N group (Figure 4A,B). The XCell analysis revealed significant increases in the numbers of myofibroblasts and SMCs and decreased numbers of epithelial cells, including stem cells, transit‐amplifying cells, Paneth cells and goblet cells (Figure 4C). inconsistent with the results of the spatial proteomic analysis, metabolic processes occurred mainly in the epithelial layer, and the downregulated pathways were related mainly to the loss of epithelial cells. We determined the relationship between NEC and upregulated myogenesis by investigating the highly expressed proteins involved in this process. Proteins expressed in myofibroblasts that are related to contraction and myofilament reprogramming during the EMT and those that are significantly activated by the EMT were detected (Figure S4A). Thus, the upregulation of myogenesis might result from the overactivation of myofibroblasts during the EMT. Additionally, substantial disruptions of the immune microenvironment were detected by GSVA, with upregulation of inflammation‐related pathways and pathways determining the immune cell fate, which might have triggered the EMT (*p *< .05; Figure 4D). The XCell analysis of immune cells also revealed significant increases in the numbers of lymphoid and myeloid cells, notably neutrophils (*p *< .05), while the numbers of dendritic cells (DCs) and B cells decreased (*p *< .05; Figure 4C). Consistent with the changes in immune cells, complement activation was characterised by increased membrane attack complexes and impaired antigen presentation with decreased expression of major histocompatibility complex (MHC) molecules (Figure S4B).

A weighted gene coexpression network analysis (WGCNA) was performed to identify the correlations between protein expression and clinical features and to further clarify the link between the EMT and adverse outcomes of NEC (Table S3). The weighted correlation analysis of the proteins revealed 20 functional modules, which are labelled with colours (Figure S4C). Intestinal stenosis after NEC was negatively correlated with the brown and pink modules (*p *< .05; Figure S4C). As proteins in those modules function to inhibit immune responses and antagonise fibrotic signalling, the decreasing of which showed relieved negative regulatory effects, the enrichment analysis of these proteins revealed the upregulated pathways in infants with stenosis. Compared with those without stenosis, infants with intestinal stenosis exhibited upregulated immune responses and inflammation‐related pathways. These pathways included primarily the RIG‐I‐like receptor signalling pathway, Th1 and Th2 cell differentiation, Th17 cell differentiation and the chemokine signalling pathway. Additionally, cell proliferation and fibrosis‐related signalling, such as the phospholipase D and apelin signalling pathways, were upregulated (Figure 4E). These findings indicate that excessive inflammatory responses and fibrosis signals, which might be resulted from fibroblast activation and ECM deposition, are related to the development of stenosis after NEC.

### 4.4 Single‐cell sequencing revealed excessive inflammation and EMT activation in epithelia and transformation of myofibroblast in infants with NEC

**FIGURE 5:**
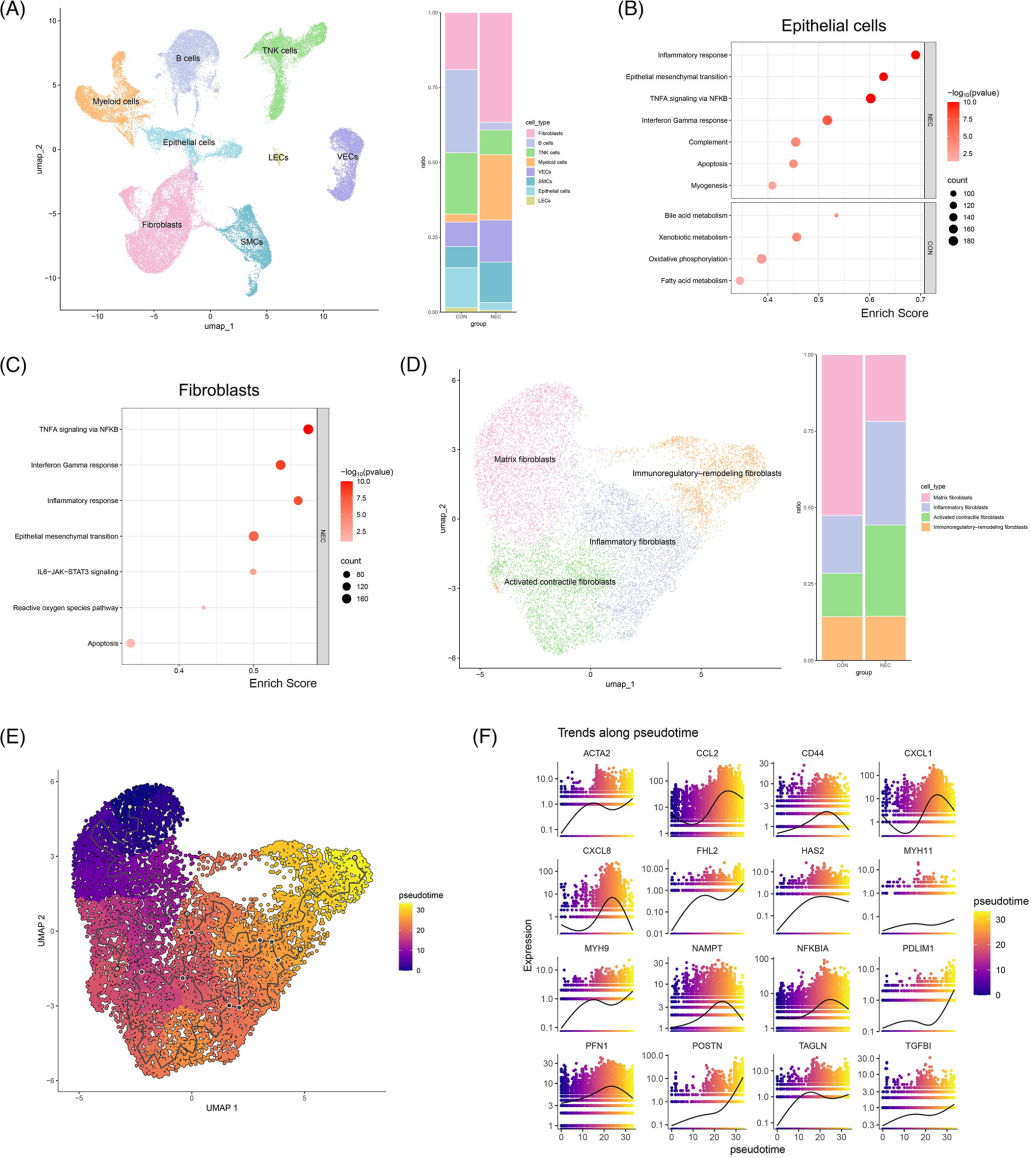
Single‐cell sequencing analysis of infants with necrotising enterocolitis (NEC). (A) Comparison of the cellular composition and percentages between the CON and NEC groups. (B) Gene set enrichment analysis (GSEA) of pathways identified in epithelial cells. (C) GSEA of pathways identified in fibroblasts. (D) Comparison of the cellular composition and percentages of fibroblasts between the CON and NEC groups. (E) Trajectory analysis of subclusters of fibroblasts. (F) Changes in differentially expressed genes determined using a pseudotime analysis.

To further validate our proteomics findings of the role of EMT in NEC and identify the specific cell subpopulations involved in these processes, we conducted single‐cell sequencing on six intestinal tissue samples (three from infants with NEC and three infants without NEC) to elucidate the primary dysfunctions and mechanisms of NEC at the cellular level. After cell filtering, identification of highly variable genes, and UMAP analysis, we identified 43 388 cells divided into nine clusters (Figure S5A). Annotation revealed clusters of fibroblasts, B cells, T/NK cells, myeloid cells, vascular endothelial cells (VECs), SMCs, epithelial cells and lymphatic endothelial cells (LECs), with distinct cellular compositions in the NEC and control groups (Figures 5A, S5B,C and Supporting Information Data 1). The proportions of fibroblasts and SMCs increased, whereas that of epithelial cells decreased, supporting our conclusion regarding the changes of the EMT in NEC (Figure 5A). GSEA revealed the downregulation of metabolic pathways and upregulation of inflammatory signalling pathways in epithelial cells. Notably, the EMT and apoptosis were significantly upregulated in epithelial cells, whereas myogenesis was increased in SMCs (Figures 5B and S5D), suggesting that excessive inflammation‐induced apoptosis in the epithelium might be correlated with EMT, potentially leading to increased muscle generation. In fibroblasts, an increased inflammatory response and upregulated EMT were observed (Figure 5C). We further investigated the EMT and its effects on NEC, by reclustering fibroblasts into four subclusters: matrix fibroblasts, inflammatory fibroblasts, activated contractile fibroblasts and immunoregulatory‐remodelling fibroblasts (Figures 5D, S5E and Supporting Information Data 2). The numbers of matrix fibroblasts, homeostatic fibroblasts in the intestine, which are characterised by ECM production, were reduced, whereas the numbers of activated contractile fibroblasts (myofibroblasts) and inflammatory fibroblasts were increased in NEC (Figures 5D and S5F). To investigate fibroblast activation and myofibroblast transformation associated with EMT‐like remodelling, we performed a pseudotime analysis (Figure 5E). The trajectory illustrated a progression from matrix fibroblasts to myofibroblasts and inflammatory fibroblasts (Figure S5G). The expression of genes associated with exceptional barrier repair, such as *ACTA2*, *TAGLN*, *MYH11*, *MYH9*, *POSTN*, *FHL2*, *TGFBI* and *HAS2*, and inflammation‐related genes, such as *CCL2, CXCL1, CXCL8* and *NFKBIA*, increased (Figure 5F). Single‐cell sequencing corroborated our proteomic findings, suggesting that EMT might be correlated with excessive inflammation, leading to decreased number of epithelial cells and increased of myofibroblasts, potentially contributing to the progression of NEC through an imbalance between damage and repair.

### 4.5 The inhibition of the EMT in animal models indicated the relationship between EMT and excessive inflammation in NEC

**FIGURE 6:**
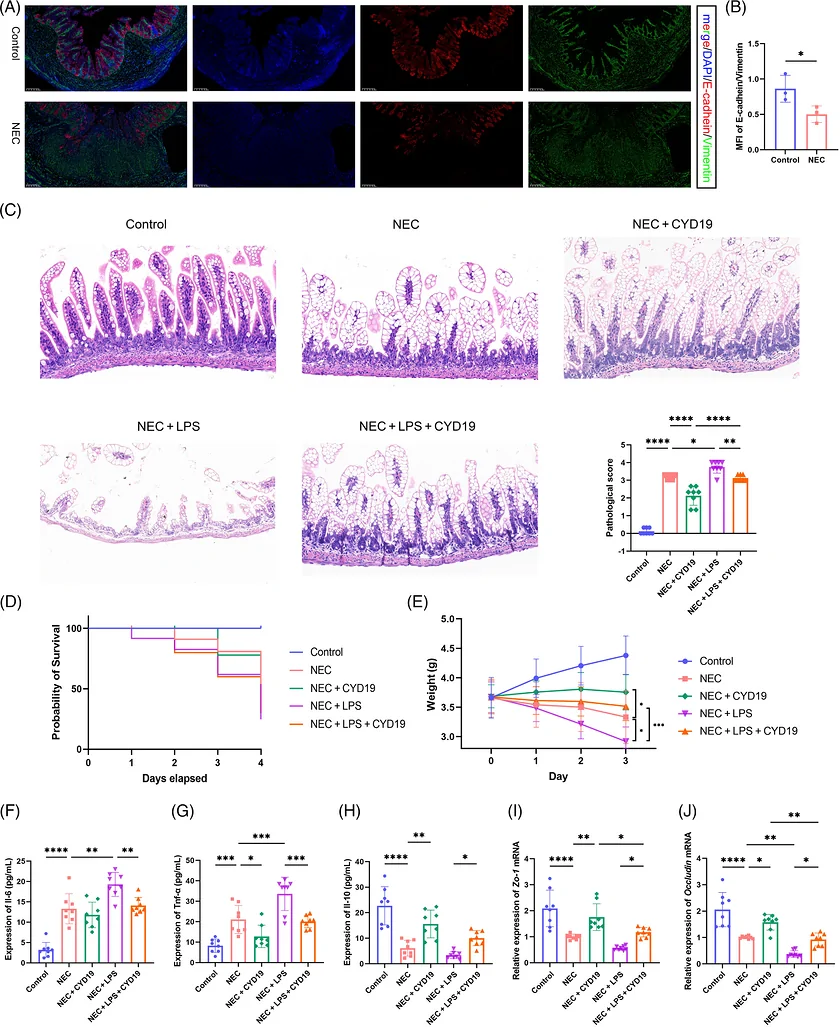
Animal experiments in necrotising enterocolitis (NEC) mice in which the epithelial–mesenchymal transition (EMT) was inhibited. (A) Immunofluorescence staining for E‐cadherin/Vimentin in intestines of NEC infants. (B) Comparison of median fluorescence intensity (MFI) for E‐cadherin/Vimentin in intestines of NEC infants (*n* = 3). (C) Haematoxylin and eosin staining and histological scoring of mouse intestines (*n* = 8). (D) Survival rate of the mice. (E) Changes in the weights of the mice during modelling (*n* = 8). (F) Expression level of Il‐6 in mouse intestines (*n* = 8). (G) Expression level of Tnf‐α in mouse intestines (*n* = 8). (H) Expression level of Il‐10 in mouse intestines (*n* = 8). (I) Relative expression of the *Zo‐1* mRNA in mouse intestines (*n* = 8). (J) Relative expression of the *Occludin* mRNA in mouse intestines (*n* = 8).

Based on the results of the multiomics analysis described above, we found that the EMT might contribute to the progression of NEC by linking excessive inflammation and barrier repair dysfunction. Since the above findings were discovered in a single sample, in order to enhance the generalisability of the conclusion, we verified the key phenomena related to EMT in other human samples. IF staining for E‐cadherin/Vimentin showed that compared with that in the control infants, the average fluorescence intensity of E‐cadherin in the intestinal tissues of NEC infants decreased, while that of vimentin increased, with a decreased ratio of E‐cadherin to vimentin (*p *< .05; Figure 6A,B). It indicated that the EMT was significantly activated in NEC infants and same results could be found in NEC mice (Figure S6A,B). To investigate whether EMT was a mediator in NEC progression or a secondary repair response after intestinal injury, mRNA level of *Cdh1*, *Vim* and *Acta2* were measured at different time points during the NEC modelling. We found *Cdh1* decreased over time, significantly at 24 h while *Vim* and *Acta2* increased and significantly at 48 h (Figure S6C). This temporal sequence indicated that EMT initiated during the acute phase of NEC, rather than as a late or purely reparative event. The early decline of *Cdh1* correlated with barrier dysfunction and delayed upregulated *Vim* and *Acta2* suggested that EMT was a progressive process, which might amplify inflammation in NEC.

To further validate the relationship between EMT in NEC progression by amplifying inflammation, we intervened the NEC mice model with LPS and inhibited EMT, subsequently. As the core transcription factor of the EMT, Snail can directly inhibit the expression of epithelial genes (especially E‐cadherin) and induce the expression of mesenchymal genes, fundamentally altering the phenotype and function of cells during the EMT. Therefore, we further treated NEC mice with CYD19, an inhibitor of Snail,^53^ to verify the role of the EMT in the pathogenesis of NEC. First, a preliminary experiment was conducted to determine whether CYD19 affected normal neonatal mice. Based on previous studies, the concentration of this inhibitor administered to neonatal mice was set at 15 mg/kg.^53^ No obvious inflammatory cell infiltration, villous degeneration, or oedema occurred in the mice after treatment with 15 mg/kg CYD19, and the pathological score did not increase (*p *> .05; Figure S6D). And inNEC mice, the expression of Snail was decreased significantly after 15 mg/kg CYD19 intervention (*p *< .05; Figure S6E). These findings suggest that 15 mg/kg CYD19 does not aggravate intestinal damage in mice and can be used for subsequent drug intervention effectively. Then, after treatment with CYD19, IF staining for E‐cadherin/Vimentin showed that the E‐cadherin/Vimentin ratio in the intestinal tissues of NEC mice increased (*p *< .05) and relative mRNA expression of *Cdh1* increased while that of *Vim* and the core transcription factor *Snail1*, *Snail2* and *Twist1* decreased (Figure S6A,B,F), suggesting that the intervention was effective and that the EMT was inhibited in NEC mice.

A further examination of the NEC phenotypic indicators was conducted after intervention of LPS and/or CYD19. Compared with those in the NEC group, whether LPS treatment is applied or not, the disordered arrangement of epithelial cells, villous degeneration, oedema, partial necrosis and shedding or even disappearance of intestinal tissue in the intervention group were significantly improved, and the pathological score decreased after treatment with CYD19 (*p *< .05) (Figure 6C), with an increased survival rate (*p *< .05; Figure 6D) and less weight loss (*p *< .05; Figure 6E), indicating that the inhibition of the EMT improved the general condition of NEC mice. The levels of inflammatory factors in intestinal tissues were detected using ELISAs and the results revealed decreased levels of the proinflammatory cytokines Il‐6 and Tnf‐α and increased levels of the anti‐inflammatory cytokine Il‐10 in NEC mice after the CYD19 intervention (*p *< .05; Figure 6F–H). These findings suggested that inflammatory cell infiltration in mice was reduced after EMT inhibition. The mRNA expression of barrier proteins was detected using qPCR, and compared with that in the NEC group, the relative mRNA expression of both *Zo‐1* (*p *> .05) and *Occludin* (*p *< .05) increased after EMT inhibition (Figure 6I,J). These results revealed that inhibiting the EMT can significantly improve the phenotype of NEC mice. Compared with those in the NEC group, mice intervened with LPS showed worse general condition, more severe infiltration of inflammatory cells and barrier damage while on this basis, blocking EMT with CYD19 could reverse the aggravation of NEC phenotype caused by LPS (Figure 6C–J), suggesting that the EMT is associated with excessive inflammation and intestinal damage in NEC progression.

### 4.6 ANXA2, RPS28, RPL23A and RPL13 may serve as tissue biomarkers associated with NEC‐related intestinal injury and blood‐based candidate biomarker serum ANXA2 showed exploratory diagnostic utility

**FIGURE 7:**
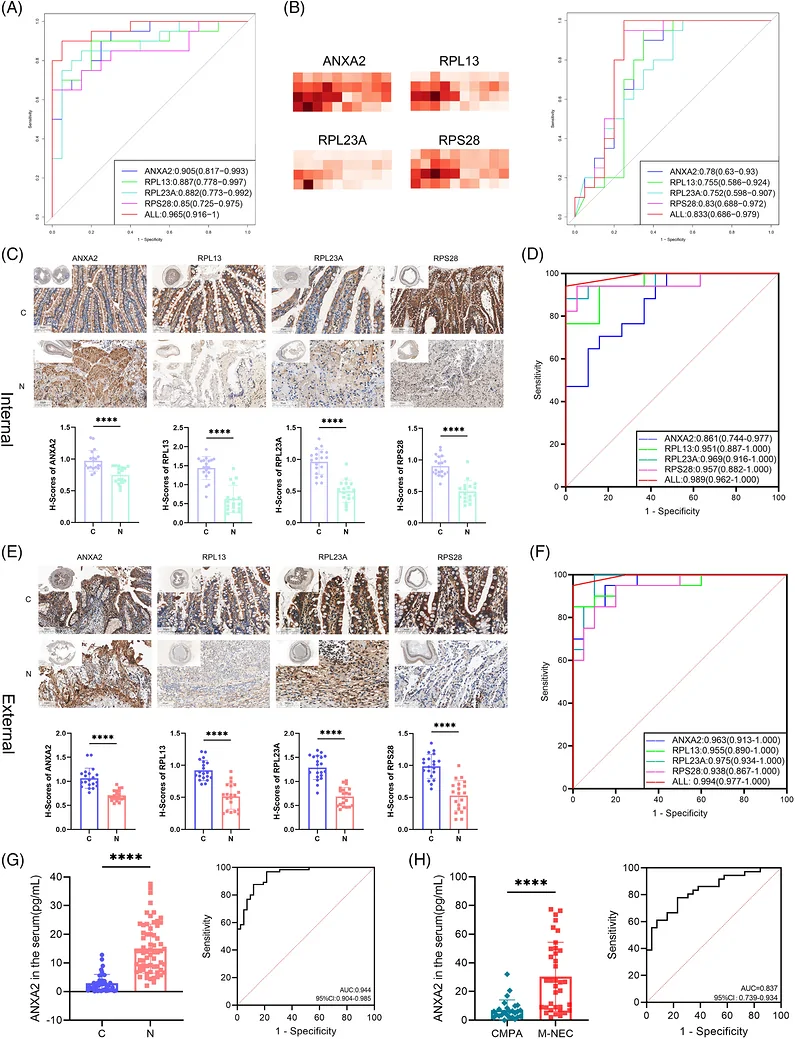
Exploration and validation of candidate biomarkers for early diagnosing necrotising enterocolitis (NEC). (A) Receiver operating characteristic (ROC) curves of biomarkers identified in the bulk proteomic analysis (*n* = 20). (B) Projection and ROC curves of biomarkers identified in the spatial proteomics analysis (*n* = 20). (C) Internal validation of biomarkers through immunohistochemical (IHC) staining (*n* = 19 patients in the C group and *n* = 17 infants in the N group). (D) ROC curves of biomarkers in the internal validation cohort. (E) External validation of biomarkers through IHC staining (*n* = 20). (F) ROC curves of biomarkers in the external validation cohort. (G) Validation and ROC curves of serum annexin A2 (ANXA2) levels between the C and N groups (*n* = 42 patients in the C group and *n* = 65 patients in the N group). (H) Validation and ROC curves of serum ANXA2 levels between the HS‐CMPA and M‐NEC groups (*n* = 26 infants in the HS‐CMPA group and *n* = 36 infants in the M‐NEC group).

We demonstrated the role of the EMT in the pathogenesis of NEC through multiomics and animal experiments described above. Based on the loss of epithelial cells, the expression of a series of proteins changed indicating that these proteins can serve as biomarkers for the early diagnosis of NEC. Therefore, receiver operating characteristic (ROC) curves were generated for each differentially expressed protein identified in the bulk proteomics, and 39 proteins with an area under the curve (AUC) of  .85 or higher were identified, which were subsequently mapped to the spatial proteomics data (Supporting Information Data 3). Due to the loss of epithelial cells, decrease in the expression of annexin A2 (ANXA2), and the ribosomal proteins L13 (RPL13), RPL23A and RPS28 were successfully mapped and selected as candidate biomarkers (Supporting Information Data 3 and Figure S7A,B). The AUCs for the bulk proteomics data were  .905,  .887,  .882 and  .85, respectively, with a combined AUC of  .965, indicating high diagnostic value (Figure 7A). For the spatial proteomics data, the AUCs were  .78,  .755,  .752 and  .83, respectively, with a combined AUC of  .833, suggesting a medium–high diagnostic value (Figure 7B).

IHC staining was conducted to validate the diagnostic value of the four proteins. In the internal validation cohort, the AUCs for ANXA2, RPL13, RPL23A and RPS28 were  .861,  .951,  .969 and  .957, respectively, with a combined AUC of  .989, indicating high diagnostic value (Figure 7C,D). In the external validation cohort, the AUCs were  .963,  .955,  .975 and  .938, respectively, with a combined AUC of  .994, also indicating strong diagnostic potential (Figure 7E,F). These results underscore the significant potential of ANXA2, RPL13, RPL23A and RPS28 to serve as tissue biomarkers associated with NEC‐related intestinal injury.

As RPL13, RPL23A and RPS28 are intracellular structural components of ribosomes and are rarely released into peripheral blood under physiological conditions, so they cannot be detected routinely in serum. Thus, only serum ANXA2 levels were detected with ELISA to explore the clinical applicability. Compared with the control group, the N group exhibited a significant increase in the serum ANXA2 level (*p *< .05), with an AUC of  .944 (Figure 7G), indicating its higher early diagnostic value for intestinal injury after NEC compared with the AUC of common clinical indicators including white blood cell (WBC), immature to total neutrophil ratio (I/T Ratio), C‐reactive protein (CRP) and procalcitonin (PCT) and existing NEC biomarkers including calprotectin (CALP), fatty acid‐binding protein 2 (FABP2), IL‐6 and IL‐8 (Figure S7C). Then, serum ANXA2 levels were compared between surgical‐NEC patients and medical‐NEC patients to verify its ability for surgical requirement prediction. No significant difference was detected within 12 h after enrolment (*p *> .05) while ANXA2 level in surgical‐NEC patients was higher than that in medical‐NEC patients within 6 h after enrolment (*p *< .05) with an AUC of  .933 (Figure S7D), indicating a relatively high value to early identify the disease progression though the sample size was small and the sampling window was narrow. To further explore the differential value of ANXA2 in neonatal intestinal diseases, we measured the serum ANXA2 levels in infants who presented mainly with bloody stools and finally diagnosed with HS‐CMPA or NEC who only received only medical treatment. We found that the serum level of ANXA2 in infants with HS‐CMPA was significantly lower than that in infants with M‐NEC and the AUC was  .837, indicating a substantial difference (*p *< .05; Figure 7H). Given the intergroup differences in baseline clinical characteristics between the M‐NEC group and HS‐CMPA group, we performed a multivariate logistic regression analysis adjusted for gestational age, birth weight, 1‐min Apgar score and serum ANXA2. Elevated serum ANXA2 was identified as an independent risk factor for M‐NEC. After adjustment for clinical covariates, the AUC of serum ANXA2 for differentiating NEC and HS‐CMPA reached  .899 (Figure S7E). These findings suggest that serum ANXA2 is a blood‐based promising biomarker for early diagnosing and distinguishing NEC from other intestinal diseases and might predict disease severity for surgical requirement within 6 h of the onset of NEC.

To explore the relationship between increased serum level and decreased intestinal level of ANXA2, the localisation of ANXA2 in the intestine of the IHC cohort was analysed. It was found that in the control group, ANXA2 was located on the cell membrane and cytoplasm of intestinal epithelial cells, while there was almost no ANXA2 expression in the interstitium and blood vessels; in the NEC group, the expression of ANXA2 in intestinal epithelium was significantly weakened, and ANXA2 was diffusely spilled from the necrotic and detached epithelium, the damaged mucosal wound surface, and could be seen spreading to the interstitium, submucosa and microvessels around, suggesting that the increase of ANXA2 in the serum of the NEC group might be due to the destruction of intestinal epithelium, resulting in the release of ANXA2 into the blood from the intestine (Figure S7F).

## 5 DISCUSSION

Early diagnosis and targeted treatment are essential for improving the prognosis of infants with NEC. However, specific and reliable investigations to elucidate pathological progression are lacking. The 2024 advancement in spatial proteomics enables the analysis of disease progression and biomarker identification using samples from adjacent normal and necrotic tissues on a slide.^20^, ^21^, ^22^ This preliminary study investigated the potential pathogenesis of NEC and identified early diagnostic biomarkers and therapeutic targets, thereby providing new insights into NEC through a spatial proteomics analysis of FFPE sections.

The induction of the layer‐specific EMT associated with an inflammatory response might be associated with the progression of NEC. Utilising spatial proteomics, we observed that epithelial inflammation induces ferroptosis and necroptosis, whereas increased inflammation and impaired repair in the submucosa facilitate progression to the muscularis layer. During this process, the EMT is activated and the phenotype of experimental NEC animals improved after intervention of the core transcription factor of EMT. These preliminary findings suggest that the EMT might play an important role in the pathogenesis of NEC as a link of excessive inflammation and intestinal damage in NEC progression. In the healthy submucosa, the EMT, in addition to the mesenchymal–epithelial transition, is vital for intestinal epithelium renewal and tissue repair.^54^, ^55^ During inflammation, the EMT is activated, and MSCs are converted into fibroblast‐like cells for reconstruction, which subsequently subsides as the inflammatory response diminishes.^54^, ^55^ However, in individuals with acute intestinal inflammation, the type II EMT characterised by inflammation and fibrosis is activated, and persistent inflammatory stimuli, such as the IL‐17A signalling pathway, induce the EMT.^56^, ^57^ The core transcription factors involved in the EMT regulate the expression of E‐cadherin and tight junction proteins (such as ZO‐1, occludin and claudin‐1), leading to a significant increase in intestinal barrier permeability and allowing luminal contents, bacteria and toxins to translocate into the submucosa, triggering local inflammation and further systemic dissemination.^58^, ^59^, ^60^ After the induction of inflammation in intestinal organoids from infants with TNF‐α and TGF‐β1, Vimentin expression is significantly upregulated in intestinal epithelial cells, whereas E‐cadherin expression is markedly downregulated, accompanied by the disruption of tight junction integrity and increased barrier permeability, providing direct evidence of the relationship between the EMT and intestinal barrier impairment.^61^ Concurrently, epithelial cells undergoing the EMT may transition from a barrier‐protective phenotype to a proinflammatory phenotype with release of proinflammatory mediators, establishing a vicious cycle of inflammatory stimulation and EMT activation.^62^, ^63^ Moreover, overstimulated epithelial cells transform into MSCs, which differentiate into fibroblasts and myofibroblasts with profibrotic and proinflammatory properties, disrupting the balance between ECM deposition and remodelling.^64^, ^65^ Continuous attack induces abnormal myofibroblast proliferation, triggering progressive fibrosis, pathological ECM deposition and scar formation, which ultimately lead to organ dysfunction,^66^, ^67^ as observed in individuals with fibrosis following inflammatory bowel disease.^68^, ^69^ During NEC healing, excessive myofibroblasts and ECMs impair repair, causing abnormal structures, fibrosis and stenosis. Targeting EMT mechanisms; signalling pathways such as the TGF‐beta, Notch and IL‐17A pathways^70^, ^71^, ^72^; and proteins and enzymes involved in ECM deposition, remodelling or myofibroblast apoptosis could represent potential NEC interventions and treatments.

Our proteomic analysis indicated that ANXA2, RPS28, RPL23A and RPL13 were potential tissue biomarkers associated with NEC‐related intestinal injury for early diagnosis of NEC. Notably, ANXA2 levels were significantly increased in serum from patients with NEC, indicating its potential release from damaged intestines to serum and possible clinical application. RPS28, RPL23A and RPL13, which are components of ribosomal proteins, are typically expressed in rapidly dividing and metabolically active cells such as intestinal epithelial cells.^73^ In infants with NEC, their reduction in the intestine might be primarily attributed to the loss of epithelial cells and NEC‐associated stressors such as ischaemia and hypoxia may further suppress the transcription of ribosomal proteins.^74^, ^75^ This decrease in the expression of ribosomal proteins impairs protein synthesis, compromising intestinal barrier renewal and repair and exacerbating cell death. Furthermore, decreased synthesis of proteins involved in nutrient metabolism leads to metabolic disruptions in infants with NEC.^76^ All of these could be potential mechanisms by which these ribosomal proteins are involved in the pathogenesis of NEC. However, the specific mechanism still requires further investigation. ANXA2, a membrane‐associated protein, is predominantly expressed in intestinal epithelial cells, including goblet cells and enterocytes, and has been recognised as a biomarker and therapeutic target in conditions such as breast cancer, colon cancer and inflammatory bowel disease.^77^, ^78^ During NEC, epithelial cell apoptosis results in the release of cellular components, reducing the intestinal tissue mass and increasing the peripheral blood levels of these proteins. ANXA2 regulates autophagy via mTORC2‐dependent pathways and endocytosis, promoting autophagy and inhibiting apoptosis.^79^, ^80^, ^81^ Thus, decreased ANXA2 expression in the intestine exacerbates apoptosis. Additionally, the ANXA2–S100A10 complex converts plasminogen to plasmin, activating matrix metalloproteinases and promoting ECM degradation.^82^, ^83^, ^84^, ^85^ Unlike previous studies,^86^, ^87^ the biomarkers we identified in FFPE intestinal tissues and validated in peripheral serum samples showed improved specificity and reliability.

Most importantly, integrating bulk proteomics, index‐case spatial proteomics, single‐cell sequencing, targeted staining and a murine intervention model, our preliminary study suggested that NEC was associated with epithelial injury, inflammatory activation and EMT‐like fibroblast/myofibroblast remodelling. The spatial proteomics data generated a hypothesis that these changes may be organised across intestinal layers. A further clinical correlation analysis based on the bulk proteomics data revealed the potential long‐term effect of the inflammation‐induced EMT on NEC infants. Additionally, we observed that the downregulation of ANXA2, RPS28, RPL23A and RPL13 expression potentially resulted from the loss of epithelial cells during apoptosis. Notably, ANXA2 levels increased in serum, indicating its release from damaged intestinal tissue and underscoring its reliability, and significant clinical application potential.

## 6 LIMITATIONS OF THIS STUDY

Due to the requirements of ethics, it is impossible to obtain completely normal patient specimens and disease controls were enrolled in this study, which may have changes in protein expression profiles and metabolic states and may cause errors. Efforts were made to ensure that the pathological morphology was relatively normal intestinal tissue without necrosis or inflammation and completely normal mice were selected as the control group in the animal model to verify our conclusions. Although multiple regions within the same tissue section were analysed for spatial proteomics, the study remains essentially a single‐patient observation, which might substantially limit the generalisability of the spatial findings. Validation of our main findings, EMT and biomarkers were made in other NEC patients or NEC mice model, which to some extent compensated for the limitations, but further multi‐centre validation with larger sample size is needed. Although Snail inhibition CYD19 were used for EMT inhibition, as Snail is involved in multiple biological pathways beyond EMT and CYD19 may have off‐target effects, the observed phenotypic improvement in NEC cannot be fully attributed to EMT inhibition alone. The contribution of non‐EMT pathways related to Snail should be paid more attention to and conditional gene knockout, lineage‐tracing experiments and organoid‐based NEC models are required for further validation. Larger, multi‐centre cohorts are needed to validated the diagnostic value of serum ANXA2 to predict disease severity or explore early risk stratification for surgical requirement in larger sample size and wider sampling window and whether serum ANXA2 can serve as a biomarker for early prediction of NEC onset requires longitudinal sampling before diagnosis, during active disease and after treatment. In addition, the efficacy of serum RPS28, RPL23A and RPL13 levels as candidate diagnostic biomarkers should be validated with more sensitive techniques, such as mass spectrometry and whether ribosomal dysfunction contributes mechanistically to NEC pathogenesis still needs further investigation.

## 7 CONCLUSION

In summary, the excessive inflammation‐associated EMT might contribute to intestinal damage in NEC progression. ANXA2, RPS28, RPL23A and RPL13 were associated with NEC‐related intestinal injury at the tissue level, while serum ANXA2 showed exploratory diagnostic utility for distinguishing NEC from available non‐NEC controls and HS‐CMPA. Further prospective, multi‐centre and mechanistic studies are required to validate these findings.

## AUTHOR CONTRIBUTIONS

All the authors contributed significantly to the study. Xiao‐Chen Liu, Guo‐Bin Liu and Chen Cheng equally shared the responsibilities, including study conception, design, sample collection, data analysis and interpretation, and the drafting, critical revision and approval of the manuscript. Xiao‐Chen Liu conducted validation of biomarkers including IHC and ELISA experiments, analysed single‐cell sequencing data and performed the animal experiments. Guo‐Bin Liu and Chen Cheng managed cohort inclusion/exclusion and collected clinical data. Jin Zhu prepared the formalin‐fixed paraffin‐embedded samples and interpreted pathological results. Fei‐Fan Chen analysed single‐cell sequencing data. Ying Ji managed the inclusion of the ELISA cohort and serum sample collection. Yu‐Ni Zhang, Xiao‐Lin Yan, Qing Wang, Xin‐Yu Li and Qing Ai participated in the validation of biomarkers and the animal experiments. Corresponding authors Yuan Shi and Yu He supervised the project, contributed to the study conception and design, analysed and interpreted data, and oversaw the critical revision and approval of the manuscript.

## CONFLICT OF INTEREST STATEMENT

The authors declare no conflicts of interest.

## ETHICS STATEMENT

This study was approved by the Ethics Committee of the Children's Hospital Affiliated with Chongqing Medical University (No. 2023‐540, 2024‐395). In the ELISA cohort, consent forms were obtained from the parents of the enrolled neonates. And this study was registered in the China Clinical Trial Center (Pid: 283287). The animal experiment was approved by the Animal Ethics Committee of Children's Hospital of Chongqing Medical University (No. CHCMU‐IACUC20240412010).

## Supporting information



SUPPORTING INFORMATION

SUPPORTING INFORMATION

SUPPORTING INFORMATION

SUPPORTING INFORMATION

SUPPORTING INFORMATION

SUPPORTING INFORMATION

SUPPORTING INFORMATION

SUPPORTING INFORMATION

SUPPORTING INFORMATION

SUPPORTING INFORMATION

SUPPORTING INFORMATION

SUPPORTING INFORMATION

SUPPORTING INFORMATION

SUPPORTING INFORMATION

## Data Availability

The proteomics raw data are available at the ProteomeXchange Consortium (https://proteomecentral.proteomexchange.org) through the iProX repository with dataset ID PXD067931. The raw single‐cell sequencing data are accessible via NCBI under BioProject ID PRJNA1327440. Additional information can be requested from the corresponding author.
