# Supplementary material for: Multiomics analyses revealed the roles of the epithelial–mesenchymal transition and novel early candidate diagnostic biomarkers in neonatal necrotising enterocolitis
Source: Clin Transl Med. 2026 Sep 15;16(9):e70793. doi: 10.1002/ctm2.70793 (PMC13578613; doi:10.1002/ctm2.70793)
Supplement: Supplementary file 1 [file CTM2-16-e70793-s003.pdf]

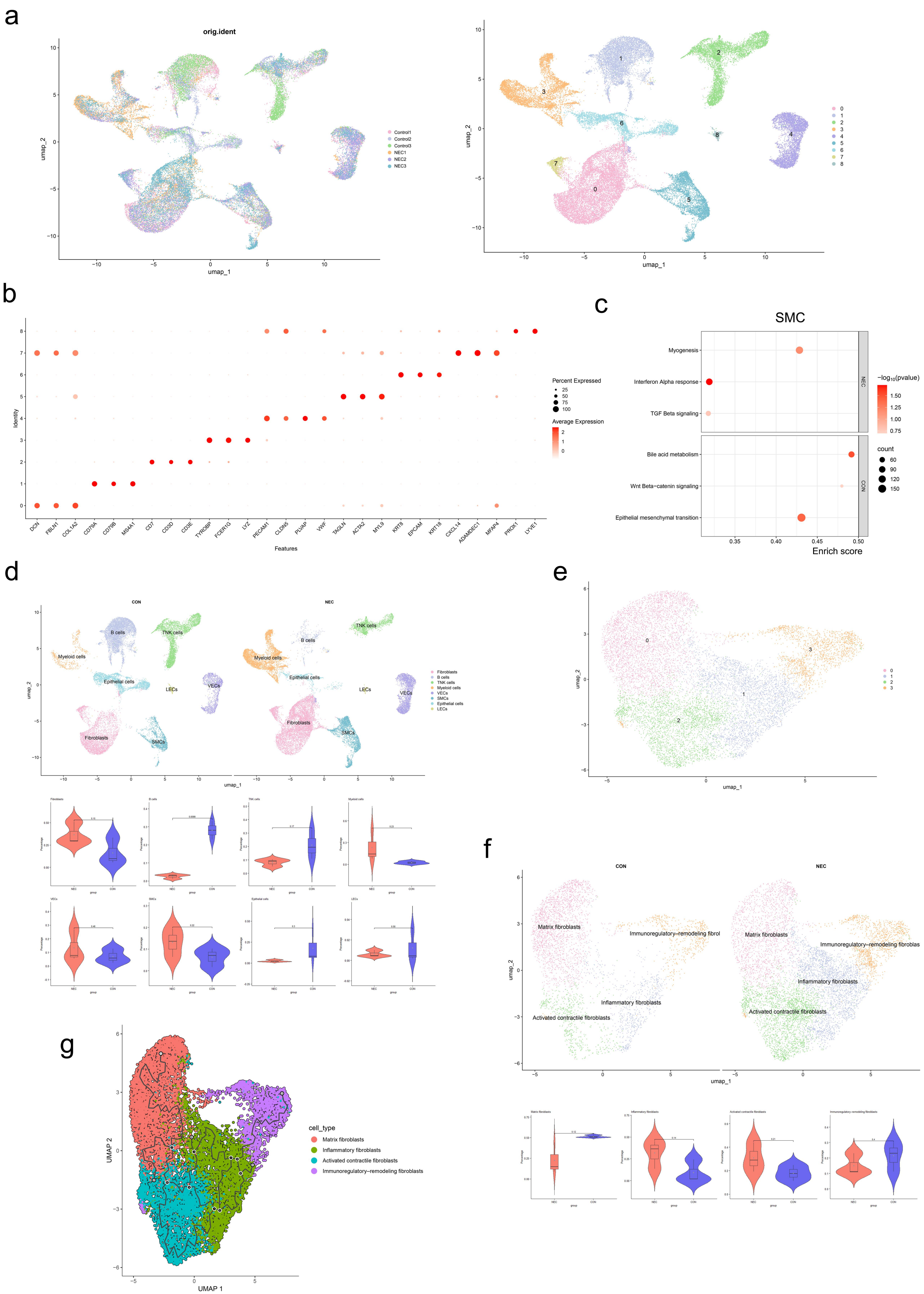
a
orig.ident
Control1
Control2
Control3
NEC1
NEC2
NEC3
umap_1
umap_2
b
Percent Expressed
Average Expression
Identity
Features
DCN FBLN1 COL1A2 CD79A CD79B MS4A1 CD7 CD3D CD3E TYROBP FCER1G LYZ PECAM1 CLDN5 PLVAP VWF TAGLN ACTA2 MYL9 KRT8 EPCAM KRT18 CXCL14 ADAMDEC1 MFAP4 PROX1 LYVE1
c
SMC
Myogenesis
Interferon Alpha response
TGF Beta signaling
Bile acid metabolism
Wnt Beta−catenin signaling
Epithelial mesenchymal transition
NEC
CON
−log10(pvalue)
count
Enrich score
d
CON
NEC
B cells
TNK cells
Myeloid cells
Epithelial cells
LECs
VECs
Fibroblasts
SMCs
e
f
Matrix fibroblasts
Immunoregulatory−remodeling fibroblasts
Inflammatory fibroblasts
Activated contractile fibroblasts
g
cell_type
Matrix fibroblasts
Inflammatory fibroblasts
Activated contractile fibroblasts
Immunoregulatory−remodeling fibroblasts
UMAP 1
UMAP 2
