# Supplementary material for: Multiomics analyses revealed the roles of the epithelial–mesenchymal transition and novel early candidate diagnostic biomarkers in neonatal necrotising enterocolitis
Source: Clin Transl Med. 2026 Sep 15;16(9):e70793. doi: 10.1002/ctm2.70793 (PMC13578613; doi:10.1002/ctm2.70793)
Supplement: Supplementary file 2 [file CTM2-16-e70793-s005.pdf]

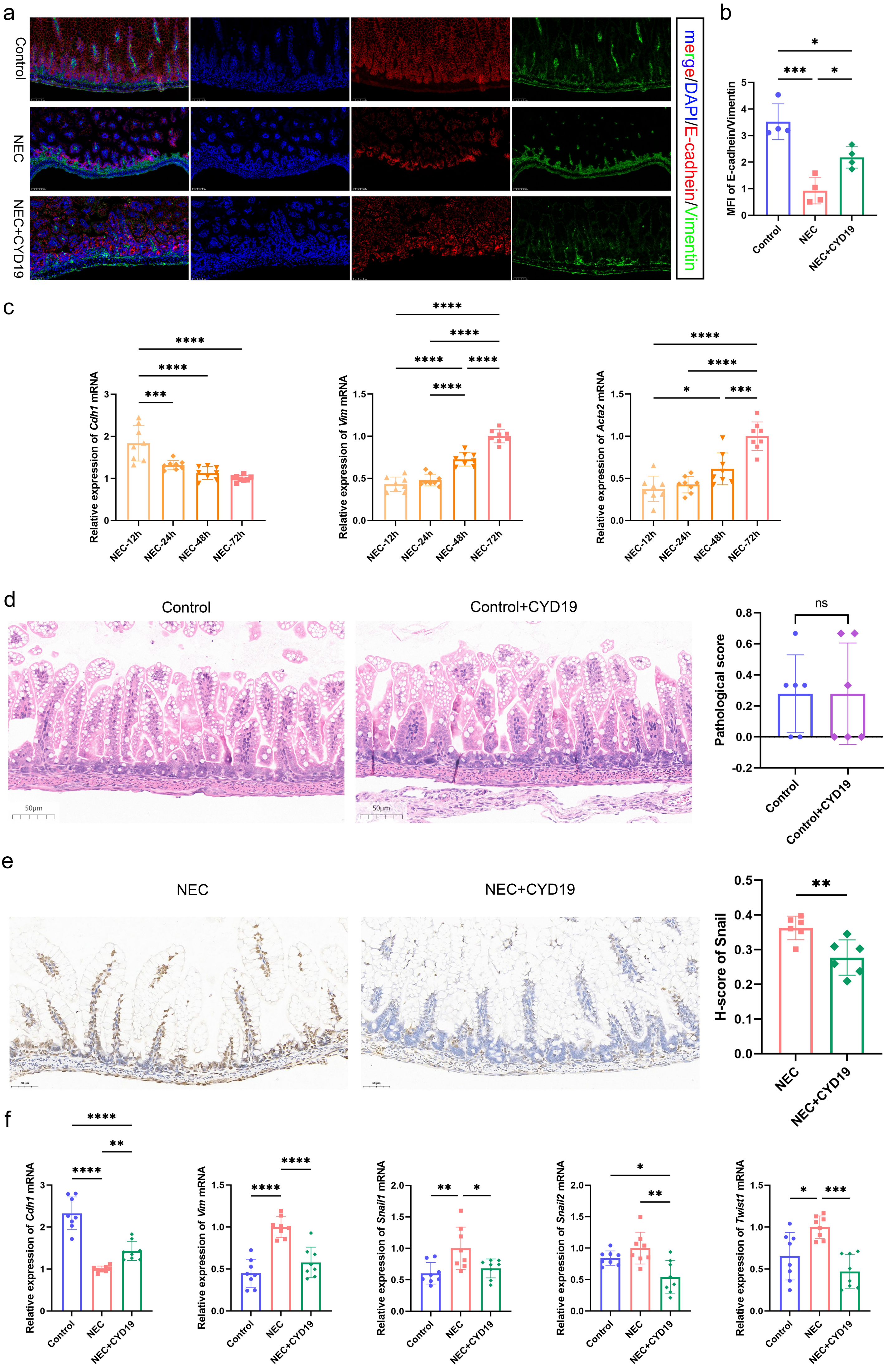

a
Control
NEC
NEC+CYD19
merge/DAPI/E-cadhein/Vimentin
b
MFI of E-cadhein/Vimentin
Control
NEC
NEC+CYD19
c
Relative expression of Cdh1 mRNA
Relative expression of Vim mRNA
Relative expression of Acta2 mRNA
NEC-12h
NEC-24h
NEC-48h
NEC-72h
d
Control
Control+CYD19
50μm
Pathological score
ns
e
NEC
NEC+CYD19
H-score of Snail
f
Relative expression of Cdh1 mRNA
Relative expression of Vim mRNA
Relative expression of Snail1 mRNA
Relative expression of Snail2 mRNA
Relative expression of Twist1 mRNA
