# Supplementary material for: Multiomics analyses revealed the roles of the epithelial–mesenchymal transition and novel early candidate diagnostic biomarkers in neonatal necrotising enterocolitis
Source: Clin Transl Med. 2026 Sep 15;16(9):e70793. doi: 10.1002/ctm2.70793 (PMC13578613; doi:10.1002/ctm2.70793)
Supplement: Supplementary file 3 [file CTM2-16-e70793-s006.pdf]

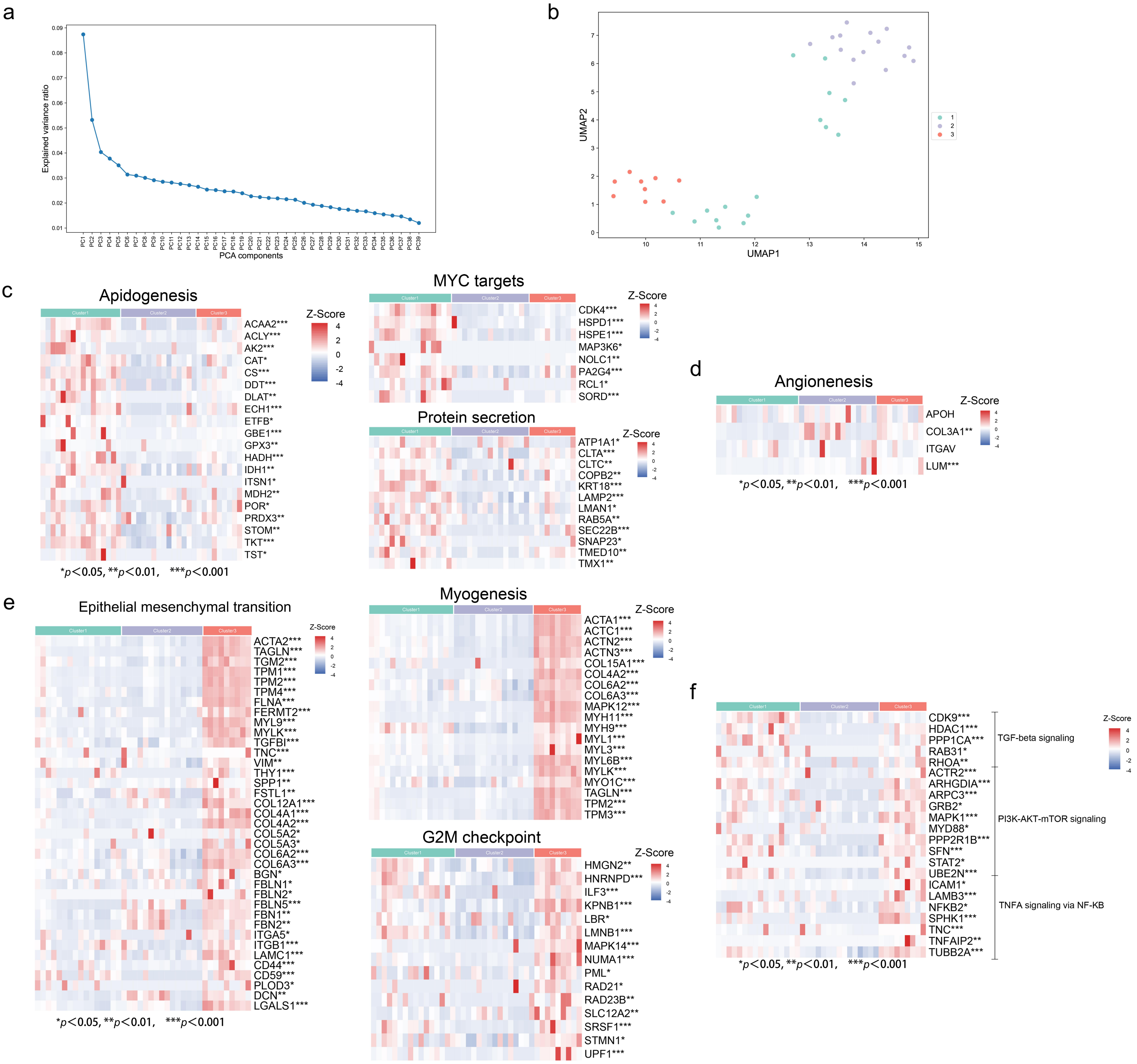

a
Explained variance ratio
PCA components
b
UMAP1
UMAP2
1
2
3
c
Apidogenesis
Z-Score
ACAA2***
ACLY***
AK2***
CAT*
CS***
DDT***
DLAT**
ECH1***
ETFB*
GBE1***
GPX3**
HADH***
IDH1**
ITSN1*
MDH2**
POR*
PRDX3**
STOM**
TKT***
TST*
*p<0.05, **p<0.01, ***p<0.001
MYC targets
Z-Score
CDK4***
HSPD1***
HSPE1***
MAP3K6*
NOLC1**
PA2G4***
RCL1*
SORD***
Protein secretion
Z-Score
ATP1A1*
CLTA***
CLTC**
COPB2**
KRT18***
LAMP2***
LMAN1*
RAB5A**
SEC22B***
SNAP23*
TMED10**
TMX1**
d
Angionenesis
Z-Score
APOH
COL3A1**
ITGAV
LUM***
*p<0.05, **p<0.01, ***p<0.001
e
Epithelial mesenchymal transition
Z-Score
ACTA2***
TAGLN***
TGM2***
TPM1***
TPM2***
TPM4***
FLNA***
FERMT2***
MYL9***
MYLK***
TGFBI***
TNC***
VIM**
THY1***
SPP1**
FSTL1**
COL12A1***
COL4A1***
COL4A2***
COL5A2*
COL5A3*
COL6A2***
COL6A3***
BGN*
FBLN1*
FBLN2*
FBLN5***
FBN1**
FBN2**
ITGA5*
ITGB1***
LAMC1***
CD44***
CD59***
PLOD3*
DCN**
LGALS1***
*p<0.05, **p<0.01, ***p<0.001
Myogenesis
Z-Score
ACTA1***
ACTC1***
ACTN2***
ACTN3***
COL15A1***
COL4A2***
COL6A2***
COL6A3***
MAPK12***
MYH11***
MYH9***
MYL1***
MYL3***
MYL6B***
MYLK***
MYO1C***
TAGLN***
TPM2***
TPM3***
G2M checkpoint
Z-Score
HMGN2**
HNRNPD***
ILF3***
KPNB1***
LBR*
LMNB1***
MAPK14***
NUMA1***
PML*
RAD21*
RAD23B**
SLC12A2**
SRSF1***
STMN1*
UPF1***
f
Z-Score
CDK9***
HDAC1***
PPP1CA***
RAB31*
RHOA**
ACTR2***
ARHGDIA***
ARPC3***
GRB2*
MAPK1***
MYD88*
PPP2R1B***
SFN***
STAT2*
UBE2N***
ICAM1*
LAMB3***
NFKB2*
SPHK1***
TNC***
TNFAIP2**
TUBB2A***
TGF-beta signaling
PI3K-AKT-mTOR signaling
TNFA signaling via NF-KB
*p<0.05, **p<0.01, ***p<0.001
