# Supplementary material for: Multiomics analyses revealed the roles of the epithelial–mesenchymal transition and novel early candidate diagnostic biomarkers in neonatal necrotising enterocolitis
Source: Clin Transl Med. 2026 Sep 15;16(9):e70793. doi: 10.1002/ctm2.70793 (PMC13578613; doi:10.1002/ctm2.70793)
Supplement: Supplementary file 4 [file CTM2-16-e70793-s009.pdf]

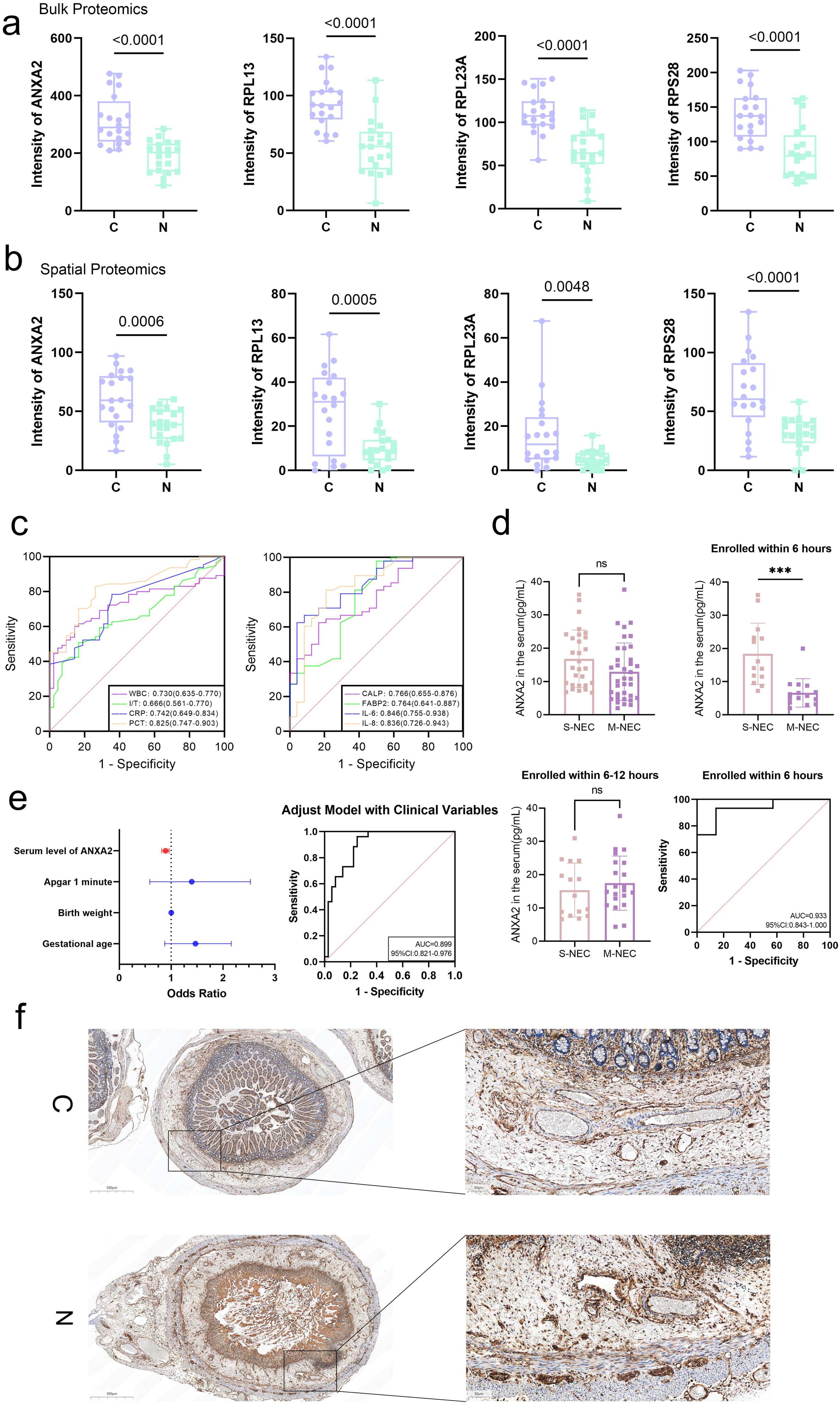
a
Bulk Proteomics
Intensity of ANXA2
<0.0001
Intensity of RPL13
<0.0001
Intensity of RPL23A
<0.0001
Intensity of RPS28
<0.0001
C
N
b
Spatial Proteomics
Intensity of ANXA2
0.0006
Intensity of RPL13
0.0005
Intensity of RPL23A
0.0048
Intensity of RPS28
<0.0001
c
Sensitivity
1 - Specificity
WBC: 0.730(0.635-0.770)
I/T: 0.666(0.561-0.770)
CRP: 0.742(0.649-0.834)
PCT: 0.825(0.747-0.903)
CALP: 0.766(0.655-0.876)
FABP2: 0.764(0.641-0.887)
IL-6: 0.846(0.755-0.938)
IL-8: 0.836(0.726-0.943)
d
Enrolled within 6 hours
ns
***
ANXA2 in the serum(pg/mL)
S-NEC
M-NEC
Enrolled within 6-12 hours
ns
Enrolled within 6 hours
AUC=0.933
95%CI:0.843-1.000
e
Serum level of ANXA2
Apgar 1 minute
Birth weight
Gestational age
Odds Ratio
Adjust Model with Clinical Variables
AUC=0.899
95%CI:0.821-0.976
f
C
N
