# Supplementary material for: Multiomics analyses revealed the roles of the epithelial–mesenchymal transition and novel early candidate diagnostic biomarkers in neonatal necrotising enterocolitis
Source: Clin Transl Med. 2026 Sep 15;16(9):e70793. doi: 10.1002/ctm2.70793 (PMC13578613; doi:10.1002/ctm2.70793)
Supplement: Supplementary file 5 [file CTM2-16-e70793-s010.pdf]

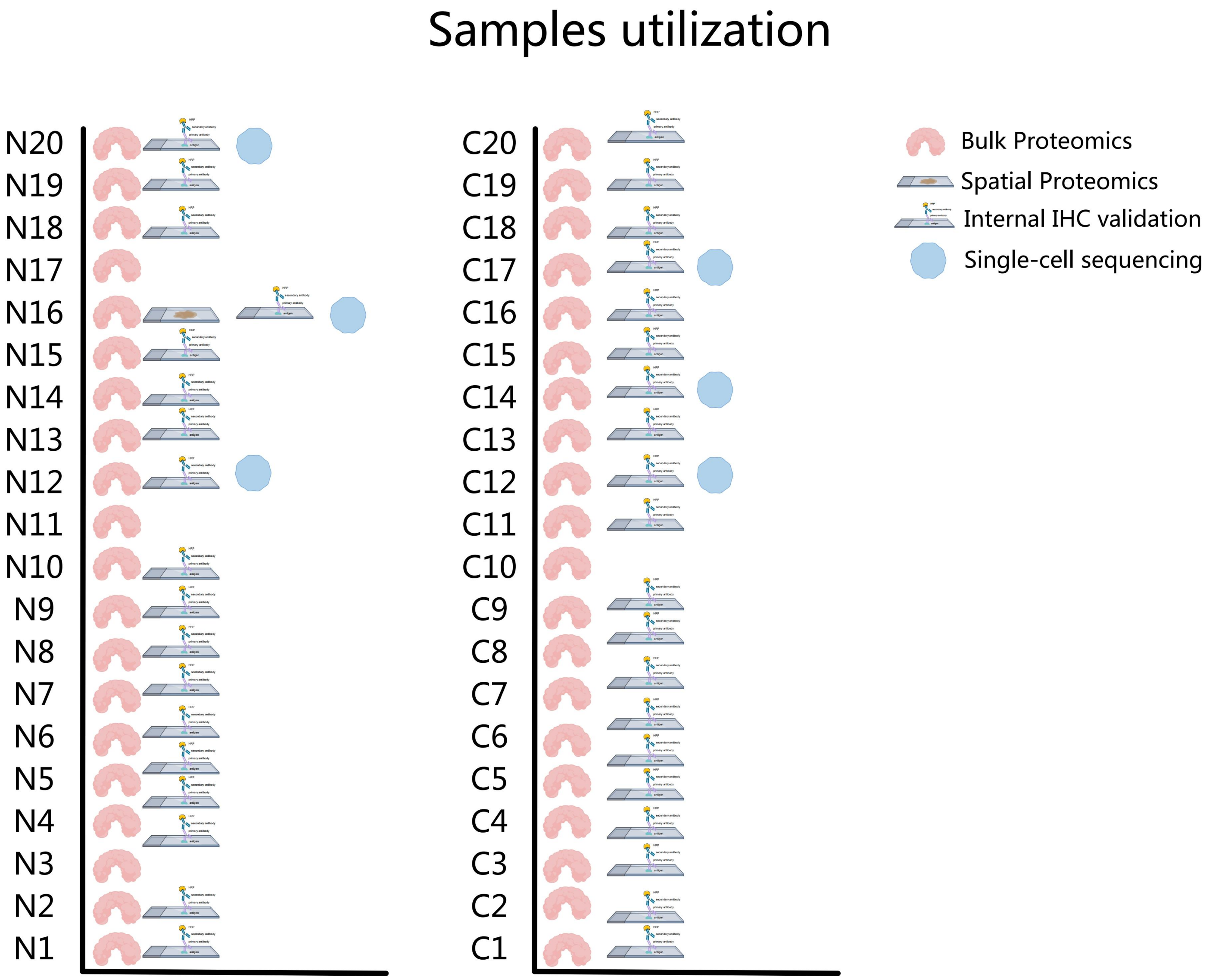
Samples utilization
N20
N19
N18
N17
N16
N15
N14
N13
N12
N11
N10
N9
N8
N7
N6
N5
N4
N3
N2
N1
C20
C19
C18
C17
C16
C15
C14
C13
C12
C11
C10
C9
C8
C7
C6
C5
C4
C3
C2
C1
Bulk Proteomics
Spatial Proteomics
Internal IHC validation
Single-cell sequencing
