# Supplementary material for: Multiomics analyses revealed the roles of the epithelial–mesenchymal transition and novel early candidate diagnostic biomarkers in neonatal necrotising enterocolitis
Source: Clin Transl Med. 2026 Sep 15;16(9):e70793. doi: 10.1002/ctm2.70793 (PMC13578613; doi:10.1002/ctm2.70793)
Supplement: Supplementary file 6 [file CTM2-16-e70793-s013.pdf]

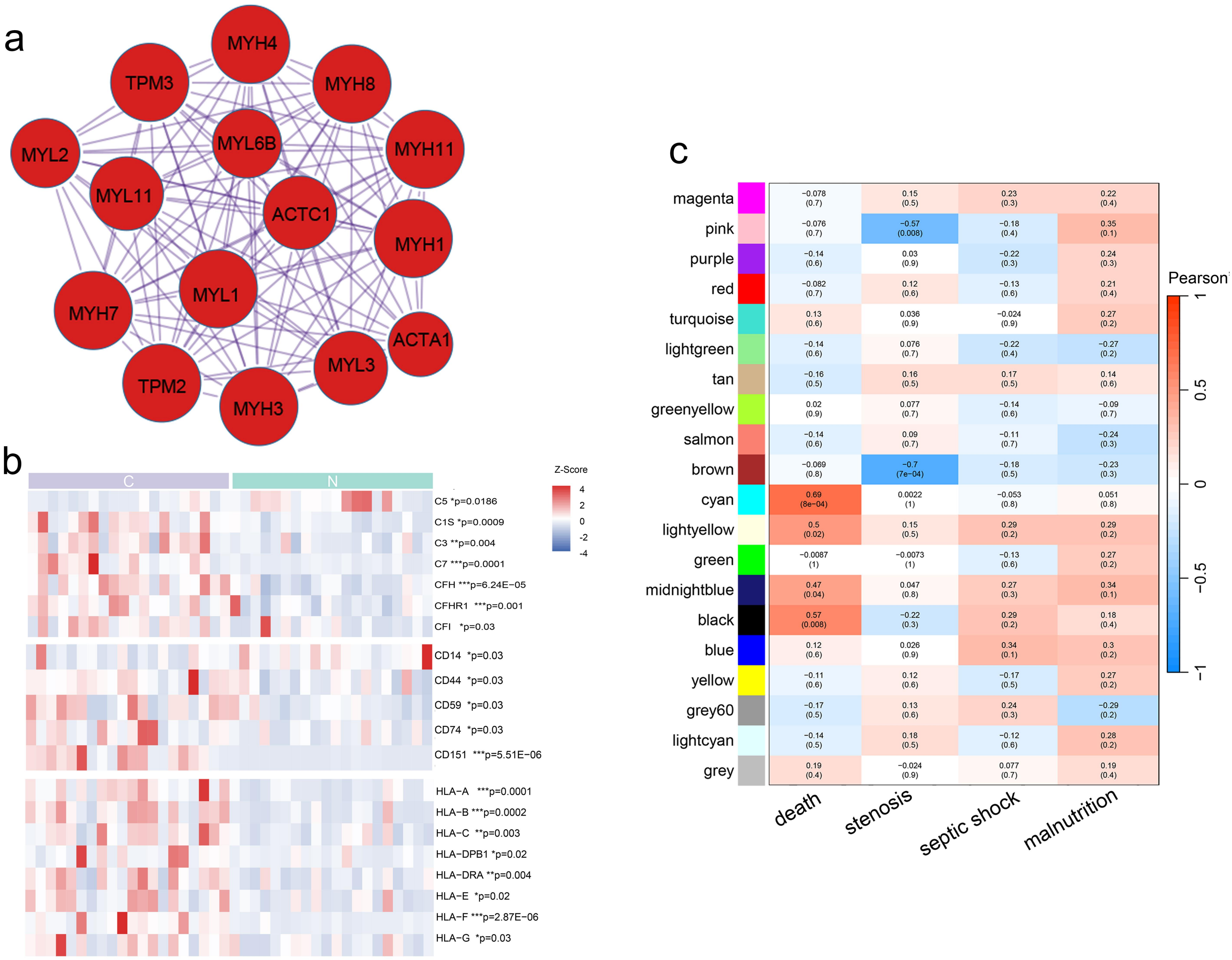
a
MYH4
TPM3
MYH8
MYL2
MYL6B
MYH11
MYL11
ACTC1
MYH1
MYH7
MYL1
ACTA1
MYL3
TPM2
MYH3
b
C
N
Z-Score
4
2
0
-2
-4
C5 *p=0.0186
C1S *p=0.0009
C3 **p=0.004
C7 ***p=0.0001
CFH ***p=6.24E−05
CFHR1 ***p=0.001
CFI *p=0.03
CD14 *p=0.03
CD44 *p=0.03
CD59 *p=0.03
CD74 *p=0.03
CD151 ***p=5.51E−06
HLA−A ***p=0.0001
HLA−B ***p=0.0002
HLA−C **p=0.003
HLA−DPB1 *p=0.02
HLA−DRA **p=0.004
HLA−E *p=0.02
HLA−F ***p=2.87E−06
HLA−G *p=0.03
c
magenta −0.078 (0.7) 0.15 (0.5) 0.23 (0.3) 0.22 (0.4)
pink −0.076 (0.7) −0.57 (0.008) −0.18 (0.4) 0.35 (0.1)
purple −0.14 (0.6) 0.03 (0.9) −0.22 (0.3) 0.24 (0.3)
red −0.082 (0.7) 0.12 (0.6) −0.13 (0.6) 0.21 (0.4)
turquoise 0.13 (0.6) 0.036 (0.9) −0.024 (0.9) 0.27 (0.2)
lightgreen −0.14 (0.6) 0.076 (0.7) −0.22 (0.4) −0.27 (0.2)
tan −0.16 (0.5) 0.16 (0.5) 0.17 (0.5) 0.14 (0.6)
greenyellow 0.02 (0.9) 0.077 (0.7) −0.14 (0.6) −0.09 (0.7)
salmon −0.14 (0.6) 0.09 (0.7) −0.11 (0.7) −0.24 (0.3)
brown −0.069 (0.8) −0.7 (7e−04) −0.18 (0.5) −0.23 (0.3)
cyan 0.69 (8e−04) 0.0022 (1) −0.053 (0.8) 0.051 (0.8)
lightyellow 0.5 (0.02) 0.15 (0.5) 0.29 (0.2) 0.29 (0.2)
green −0.0087 (1) −0.0073 (1) −0.13 (0.6) 0.27 (0.2)
midnightblue 0.47 (0.04) 0.047 (0.8) 0.27 (0.3) 0.34 (0.1)
black 0.57 (0.008) −0.22 (0.3) 0.29 (0.2) 0.18 (0.4)
blue 0.12 (0.6) 0.026 (0.9) 0.34 (0.1) 0.3 (0.2)
yellow −0.11 (0.6) 0.12 (0.6) −0.17 (0.5) 0.27 (0.2)
grey60 −0.17 (0.5) 0.13 (0.6) 0.24 (0.3) −0.29 (0.2)
lightcyan −0.14 (0.5) 0.18 (0.5) −0.12 (0.6) 0.28 (0.2)
grey 0.19 (0.4) −0.024 (0.9) 0.077 (0.7) 0.19 (0.4)
death
stenosis
septic shock
malnutrition
Pearson
1
0.5
0
−0.5
−1
