# Supplementary material for: Multiomics analyses revealed the roles of the epithelial–mesenchymal transition and novel early candidate diagnostic biomarkers in neonatal necrotising enterocolitis
Source: Clin Transl Med. 2026 Sep 15;16(9):e70793. doi: 10.1002/ctm2.70793 (PMC13578613; doi:10.1002/ctm2.70793)
Supplement: Supplementary file 7 [file CTM2-16-e70793-s014.pdf]

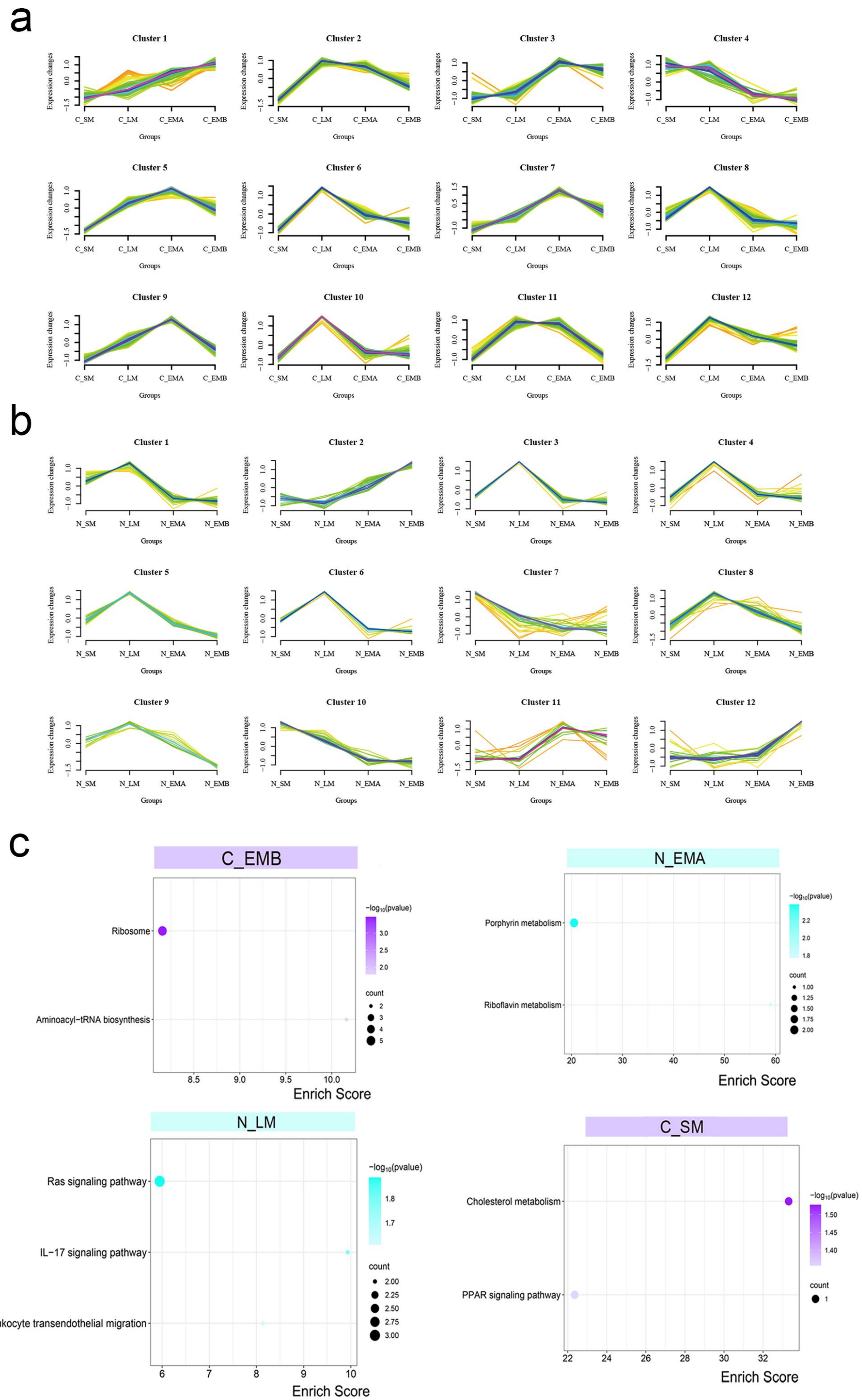

a
Cluster 1
Cluster 2
Cluster 3
Cluster 4
Cluster 5
Cluster 6
Cluster 7
Cluster 8
Cluster 9
Cluster 10
Cluster 11
Cluster 12
Expression changes
C_SM
C_LM
C_EMA
C_EMB
Groups
b
Cluster 1
Cluster 2
Cluster 3
Cluster 4
Cluster 5
Cluster 6
Cluster 7
Cluster 8
Cluster 9
Cluster 10
Cluster 11
Cluster 12
Expression changes
N_SM
N_LM
N_EMA
N_EMB
Groups
c
C_EMB
Ribosome
Aminoacyl−tRNA biosynthesis
−log10(pvalue)
count
Enrich Score
N_EMA
Porphyrin metabolism
Riboflavin metabolism
−log10(pvalue)
count
Enrich Score
N_LM
Ras signaling pathway
IL−17 signaling pathway
Leukocyte transendothelial migration
−log10(pvalue)
count
Enrich Score
C_SM
Cholesterol metabolism
PPAR signaling pathway
−log10(pvalue)
count
Enrich Score
