# Supplementary material for: Multiomics analyses revealed the roles of the epithelial–mesenchymal transition and novel early candidate diagnostic biomarkers in neonatal necrotising enterocolitis
Source: Clin Transl Med. 2026 Sep 15;16(9):e70793. doi: 10.1002/ctm2.70793 (PMC13578613; doi:10.1002/ctm2.70793)
Supplement: Supplementary file 8 [file CTM2-16-e70793-s001.docx]

|  | Proteomics cohort | | | | External validation IHC cohort | | | | | ELISA cohort | | | | | | | |
| --- | --- | --- | --- | --- | --- | --- | --- | --- | --- | --- | --- | --- | --- | --- | --- | --- | --- |
|  | **Group C**  **（n=20）** | **Group N**  **（n=20）** | ***Z/t/χ^2^*** | ***P*** | **Group C**  **（n=20）** | **Group N**  **（n=20）** | ***Z/t/χ^2^*** | | ***P*** | **Group C**  **（n=42）** | **Group N**  **（n=65）** | **Group CMPA**  **(n=26)** | ***^a^Z/t/χ^2^*** | ***^a^P*** | ***^b^Z/t/χ^2^*** | ***^b^P*** |  |
| Male, %(n) | 65.00(13) | 65.00(13) | 0.000 | 1.000 | 45.00(9) | 55.00(11) | 0.400 | | 0.527 | 38.10(16) | 52.30(34) | 53.80(14) | 2.070 | 0.150 | 0.018 | 0.894 |  |
| gestational age, day | 38.71(37.64, 39.32) | 28.79(27.86, 33.11) | -5.332 | 0.000 | 36.96±2.778 | 31.98±5.189 | 3.777 | | 0.001 | 34.74±2.423 | 34.68±2.862 | 37.26±1.743 | 0.109 | 0.913 | -4.009 | 0.000 |  |
| Birth weight, g | 3129.00±369.123 | 1307.25±438.584 | 14.212 | 0.000 | 2696.40±872.889 | 1950.75±903.027 | 2.655 | | 0.012 | 2249.55±633.080 | 2211.95±686.524 | 2966.35±476.767 | 0.285 | 0.776 | -4.577 | 0.000 |  |
| Apgar 1 minute | 10(10, 10) | 7.50(6.00, 9.00) | -4.941 | 0.000 | 10.00 (9.00,10.00) | 9.00(7.00, 9.50) | -2.253 | | 0.024 | 9.00(8.00, 10.00) | 9.00(9.00,10.00) | 10.00(9.00, 10.00) | -0.755 | 0.450 | -2.465 | 0.014 |  |
| Apgar 5 minutes | 10(10, 10) | 9.00(8.00, 10.00) | -3.901 | 0.002 | 10.00 (1.00,10.00) | 9.00(7.50, 10.00) | -2.679 | | 0.007 | 10.00(9.00,10.00) | 10.00(10.00,10.00) | 10.00(10.00,10.00) | -0.694 | 0.488 | -1.732 | 0.083 |  |
| Age of surgery, day | 15.67(7.64,33.43) | 24.50(10.75, 33.50) | -0.921 | 0.357 | 2.86(1.77,9.26) | 16.02(6.11, 25.78) | -3.381 | | 0.001 | / | 15.50(4.25,23.50) | / | / | / | / | / |  |
| Postmenstrual age of surgery, week | 40.57(39.32, 44.21) | 33.26(31.67, 35.41) | -5.221 | 0.000 | 37.98(36.07,39.52) | 34.65(31.61, 38.22) | -2.435 | | 0.015 | / | 37.56(33.57,39.82) | / | / | / | / | / |  |
| Fasting course, day | 4.00(3.00, 5.00) | 11.00(8.00, 13.50) | -3.814 | 0.000 | 9.10±5.088 | 10.00±6.104 | -0.507 | | 0.615 | / | 6.00(4.00,9.75) | / | / | / | / | / |  |
| Decompression course, day | 4.00(3.00, 5.00) | 8.00(6.00, 10.75) | -3.303 | 0.001 | 8.15±5.706 | 8.50±5.021 | -0.206 | | 0.838 | / | 6.00(4.00,9.00) | / | / | / | / | / |  |
| Antibiotic course, day | 10.10±3.493 | 16.22±6.916 | -3.498 | 0.001 | 15.70±8.272 | 15.10±9.553 | -0.212 | | 0.833 | / | 30.48(23.45,64.31) | / | / | / | / | / |  |
| Hospital stays after surgery, day | 11.00(7.00, 12.75) | 48.50(26.00, 99.50) | -3.901 | 0.000 | 21.00(13.25,34.25) | 39.50(17.50, 75.75) | -1.962 | | 0.05 | / | 30.48(23.45,64.31) | / | / | / | / | / |  |
| Hospital stays, day | 13.00(10.25, 14.75) | 60.50(30.75, 116.50) | -4.984 | 0.000 | 25.00(16.00,35.25) | 39.50(17.75, 85.00) | -2.002 | | 0.045 | 18.00(11.75,26.25) | 33.00(22.50,61.50) | 10.00(7.00,15.00) | -5.051 | 0.000 | -5.746 | 0.000 |  |
| ^#^Only those infants with surgical treatment were compared. ^a^ Comparation of Group C and N. ^b^ Comparation of Group M-NEC and CMPA. | | | | | | | |  |  |  |  |  |  |  |  |  |  |

Table S2. Clinical features of the infants enrolled in the proteomics cohorts.
