# Supplementary material for: Multiomics analyses revealed the roles of the epithelial–mesenchymal transition and novel early candidate diagnostic biomarkers in neonatal necrotising enterocolitis
Source: Clin Transl Med. 2026 Sep 15;16(9):e70793. doi: 10.1002/ctm2.70793 (PMC13578613; doi:10.1002/ctm2.70793)
Supplement: Supplementary file 9 [file CTM2-16-e70793-s002.docx]

|  | **Group C** | **Group N** | ***Z/t/χ^2^*** | ***P*** |
| --- | --- | --- | --- | --- |
| **Basic information** |  |  |  |  |
| Male, %(n) | 65.00(13) | 65.00(13) | 0.000 | 1.000 |
| gestational age, day | 38.71(37.64, 39.32) | 28.79(27.86, 33.11) | -5.332 | 0.000 |
| Birth weight, g | 3129.00±369.123 | 1307.25±438.584 | 14.212 | 0.000 |
| Apgar 1 minute | 10(10, 10) | 7.50(6.00, 9.00) | -4.941 | 0.000 |
| Apgar 5 minutes | 10(10, 10) | 9.00(8.00, 10.00) | -3.901 | 0.002 |
| **Risk factors** |  |  |  |  |
| Caesarean section, %(n) | 30.00(6) | 25.00(5) | 0.125 | 0.723 |
| PROM, %(n) | 5.00(1) | 30.00(6) | 2.711 | 0.096 |
| Amniotic fluid contamination, %(n) | 5.00(1) | 5.00(1) | 0.000 | 1.000 |
| Intrauterine distress, %(n) | 0.00(0) | 10.00(2) | 0.526 | 0.468 |
| Hypertension, %(n) | 0.00(0) | 10.00(2) | 0.526 | 0.468 |
| GDM, %(n) | 0.00(0) | 30.00(6) | 4.902 | 0.027 |
| Chorioamnionitis, %(n) | 0.00(0) | 10.00(2) | 0.526 | 0.468 |
| Glucocorticoids before delivery, %(n) | 0.00(0) | 75.00(15) | 24.000 | 0.000 |
| Antibiotic before delivery, %(n) | 0.00(0) | 35.00(7) | 0.6234 | 0.013 |
| **Outcomes** |  |  |  |  |
| Death, %(n) | / | 20.00(4) | / | / |
| Septic shock, %(n) | / | 40.00(8) | / | / |
| Intestinal stenosis, %(n)* | / | 33.3(5) | / | / |
| Malnutrition at 1 year old, %(n)* | / | 60.00(12) | / | / |
| **PROM: premature rupture of fetal membranes; Gestational diabetes mellitus: GDM;** | | | | |

Table S3. Clinical features of the infants enrolled in the proteomics cohorts.

*Four infants died after surgery and one infant was lost of follow-up.
