# Supplementary material for: Multiomics analyses revealed the roles of the epithelial–mesenchymal transition and novel early candidate diagnostic biomarkers in neonatal necrotising enterocolitis
Source: Clin Transl Med. 2026 Sep 15;16(9):e70793. doi: 10.1002/ctm2.70793 (PMC13578613; doi:10.1002/ctm2.70793)
Supplement: Supplementary file 10 [file CTM2-16-e70793-s004.docx]

**Figure S1.** **Sample utilized in this study.**

**Figure S2. Proteins participate in distinct pathways within each cluster.** (a) An elbow curve illustrating heterogeneity during dimensionality reduction and PCA components of 3 was chosen for further analysis. (b) Graph-based clustering algorithm for dimensionality reduction. (c) Heatmap of proteins associated with pathways in cluster 1. (d) Heatmap of proteins related to pathways in cluster 2. (e) Heatmap of proteins linked to pathways in cluster 3. (f) Heatmap of proteins involved in regulatory pathways.

**Figure S3. Spatial proteomic analysis of differences between each subgroup in the two groups.** (a) Protein clusters exhibiting concurrent alterations in the C groups. (b) Protein clusters exhibiting concurrent alterations in the N groups. (c) KEGG pathway enrichment analysis of regulated pathways within each subgroup.

**Figure S4. Results of the bulk proteomic analysis of NEC dysfunction.** (a) Highly expressed proteins involved in myogenesis. (b) Heatmap displaying immune molecules, complement molecules, MHC molecules, and CD molecules identified in the C and N groups. (c) Identification of functional modules in the weighted correlation analysis of an adverse prognosis in the N group.

**Figure S5.** **Single-cell sequencing analysis.** (a) Visualization of samples and clusters after UMAP. (b) Bubble plot of cluster annotations. (c) GSEA of pathways identified in SMCs. (d) Clustering comparison between the CON and NEC groups after UMAP. (e) Subcluster identification within fibroblasts after UMAP. (f) Subcluster comparison of fibroblasts between the CON and NEC groups after UMAP. (g) Trajectory analysis of subclusters within fibroblasts after UMAP.

**Figure S6. Animal experiments in NEC mice in which the EMT was inhibited.** (a) Immunofluorescence staining for E-cadherin/Vimentin in mouse intestines. (b) Comparation of median fluorescence intensity (MFI) for E-cadherin/Vimentin in mouse intestines (n=4). (c) Relative expression of the *Cdh1*, *Vim* and *Acta2* in mice intestines at different time points during the NEC modelling (n=8). (d) Haematoxylin‒eosin staining and histological scoring of normal mouse intestines were performed to determine the effect of CYD19 (n=6). (e) Immunohistochemistry for Snail after CYD19 intervention in NEC mice (n=6). (f) Relative expression of the *Cdh1*, *Vim*, *Snail1*, *Snail2* and *Twist1* in NEC mice intestines after CYD19 intervention (n=8).

**Figure S7. Exploration and validation of candidate diagnostic biomarkers.** (a) Differences in the expression of biomarkers between the two groups based on the bulk proteomics analyses (n=20). (b) Differences in the expression of biomarkers between the two groups based on the spatial proteomics analyses (n=20). (c) ROC curves of common clinical indicators (n=42 in C group and n=65 in N group) and biomarkers (n=24 in C group and n=48 in N group) for discrimination between C and N group. (d) Comparison of serum ANXA2 levels between the surgical NEC (S-NEC) and medical NEC (M-NEC) groups enrolled within 6 hours (n=14 in S-NEC group and n=15 in M-NEC group) and within in 12 hours (n=15 in S-NEC group and n=21 in M-NEC group). (e) Forest plot of clinical variables and serum ANXA2 after logistic regression in the HS-CMPA and M-NEC groups. (f) IHC staining of ANXA2 in C and N groups.

**Supplementary Data 1. Cellular markers of the single-cell sequencing annotation.**

**Supplementary Data 2. Cellular markers of the single-cell sequencing annotation in the fibroblast clusters.**

**Supplementary Data 3. The AUCs of the ROC curve of the differentially expressed proteins in the bulk and spatial proteomics analyses.**
