# Supplementary material for: Multiomics analyses revealed the roles of the epithelial–mesenchymal transition and novel early candidate diagnostic biomarkers in neonatal necrotising enterocolitis
Source: Clin Transl Med. 2026 Sep 15;16(9):e70793. doi: 10.1002/ctm2.70793 (PMC13578613; doi:10.1002/ctm2.70793)
Supplement: Supplementary file 11 [file CTM2-16-e70793-s011.docx]

| Table S1. Primers used in RT-qPCR. | |
| --- | --- |
| Gene | Primers (5' →3') |
| *β-actin*  （mouse） | Forward: CATCCGTAAAGACCTCTATGCCAAC  Reverse: ATGGAGCCACCGATCCACA |
| *Cdh1*  (Mouse) | Forward: CTCCAGTCATAGGGAGCTGTC  Reverse: TCTTCTGAGACCTGGGTACAC |
| *Vim*  (Mouse) | Forward: GAGATCGCCACCTACAGGAA  Reverse: GGTGTCAACCAGAGGAAGTGA |
| *Acta2*  (Mouse) | Forward: GACAATGGCTCTGGGCTCTGTA  Reverse: TTTGGCCCATTCCAACCATTA |
| *Snail1*  (Mouse) | Forward: TTCACCTTCCAGCAGCCCTA  Reverse: AGAGAGTCCCAGATGAGGGT |
| *Snail2*  (Mouse) | Forward: TGTATGGACATCGTCGGCAG  Reverse: GACTTACACGCCCCAAGGAT |
| *Twist1*  (Mouse) | Forward: GAACACTCGTTTGTGTCCCC  Reverse: GCCAGTTTGATCCCAGCGTT |
| *Zo-1*  (mouse) | Forward: CAGAGCCCTCCGATCATTCC  Reverse: TCTTCCACTGCTTTGGGTGG |
| *Occludin* (mouse) | Forward: GGCAAGCGATCATACCCAGA  Reverse: GCTGCCTGAAGTCATCCACA |
